# Investigating low intensity focused ultrasound pulsation in anhedonic depression—A randomized controlled trial

**DOI:** 10.3389/fnhum.2025.1478534

**Published:** 2025-03-24

**Authors:** Natalie M. Rotstein, Zachary D. Cohen, Amelia Welborn, Tomislav D. Zbozinek, Samir Akre, Keith G. Jones, Kaylee E. Null, Jillian Pontanares, Katy L. Sanchez, Demarko C. Flanagan, Sabrina E. Halavi, Evan Kittle, Mason G. McClay, Alex A. T. Bui, Katherine L. Narr, Robert C. Welsh, Michelle G. Craske, Taylor P. Kuhn

**Affiliations:** ^1^Department of Psychiatry and Biobehavioral Sciences, University of California, Los Angeles, Los Angeles, CA, United States; ^2^Department of Psychology, University of Arizona, Tucson, AZ, United States; ^3^Medical & Imaging Informatics Group, University of California, Los Angeles, Los Angeles, CA, United States; ^4^Department of Psychology, University of California, Los Angeles, Los Angeles, CA, United States; ^5^Department of Neurology, University of California, Los Angeles, Los Angeles, CA, United States

**Keywords:** anhedonia, anhedonic depression, major depressive disorder, low intensity focused ultrasound pulsation (LIFUP), transcranial focused ultrasound (tFUS), digital phenotyping, neuroimaging

## Abstract

**Introduction:**

Anhedonic depression is a subtype of depression characterized by deficits in reward processing. This subtype of depression is associated with higher suicide risk and longer depressive episodes, underscoring the importance of effective treatments. Anhedonia has also been found to correlate with alterations in activity in several subcortical regions, including the caudate head and nucleus accumbens. Low intensity focused ultrasound pulsation (LIFUP) is an emerging technology that enables non-invasive stimulation of these subcortical regions, which were previously only accessible with surgically-implanted electrodes.

**Methods:**

This double-blinded, sham-controlled study aims to investigate the effects of LIFUP to the left caudate head and right nucleus accumbens in participants with anhedonic depression. Participants in this protocol will undergo three sessions of LIFUP over the span of 5–9 days. To investigate LIFUP-related changes, this 7-week protocol collects continuous digital phenotyping data, an array of self-report measures of depression, anhedonia, and other psychopathology, and magnetic resonance imaging (MRI) before and after the LIFUP intervention. Primary self-report outcome measures include Ecological Momentary Assessment, the Positive Valence Systems Scale, and the Patient Health Questionnaire. Primary imaging measures include magnetic resonance spectroscopy and functional MRI during reward-based tasks and at rest. Digital phenotyping data is collected with an Apple Watch and participants' personal iPhones throughout the study, and includes information about sleep, heart rate, and physical activity.

**Discussion:**

This study is the first to investigate the effects of LIFUP to the caudate head or nucleus accumbens in depressed subjects. Furthermore, the data collected for this protocol covers a wide array of potentially affected modalities. As a result, this protocol will help to elucidate potential impacts of LIFUP in individuals with anhedonic depression.

## 1 Introduction

Anhedonic depression is a subtype of depression characterized by an array of deficits in reward processing, including reduced enjoyment of activities and rewarding events (Serretti, 2023). The presence of anhedonia in patients with major depressive disorder is associated with longer depressive episodes, greater illness severity, and increased suicidality (Gabbay et al., 2015; Ducasse et al., 2018; Auerbach et al., 2022). In addition to its association with worsened illness prognosis, anhedonia has been found to respond less reliably than negative affective symptoms to standard psychotherapeutic and psychiatric treatments for depression (Dunn et al., 2020; Craske et al., 2024). Anhedonia's unreliable response to treatment is especially problematic given that prior analyses of patient perspectives has found that many patients view the return of positive affect to be a critically important factor in recovery from depression (Zimmerman et al., 2006; Cummergen et al., 2022). This demonstrates the critical need for improved, targeted treatments for anhedonic symptoms in patients with anhedonic depression.

Low intensity focused ultrasound pulsation (LIFUP) is an emerging brain stimulation technique that shows potential to stimulate subcortical brain regions non-invasively. Preliminary data have demonstrated that when administered within the FDA safety guidelines for ultrasound in soft tissue, LIFUP is capable of reversibly modulating neural activity without causing damage to tissue (Pasquinelli et al., 2019). Furthermore, it is able to modulate deep brain regions with high spatial specificity (Kuhn et al., 2023; Chou et al., 2024; Pellow et al., 2024; Riis et al., 2024a,b). While surgical deep brain stimulation (DBS) to reward-related subcortical regions, such as the caudate and nucleus accumbens, has been shown to be efficacious for the treatment of anhedonic depression, it is highly invasive, requiring major brain surgery that poses significant risk of complications (Fenoy and Simpson, 2014; Figee et al., 2022). This makes LIFUP an intervention of interest for anhedonic symptoms, as it is able to non-invasively stimulate these same deep brain regions in a non-invasive manner.

Investigating LIFUP in Anhedonic Depression (ILIAD) is a complementary interventional study to a broader observational study: Operationalizing Digital PhenoTyping in the Measurement of Anhedonia (OPTIMA). These two studies are distinct and have only partially overlapping subject populations; while OPTIMA recruits participants across the spectrum of anhedonia severity, ILIAD only enrolls participants with high levels of anhedonia. Additionally, ILIAD recruits from both the OPTIMA subject pool and from external sources. However, many elements of the two studies are harmonized in order to facilitate potential merging of data. Both of these studies are performed in the Wellcome Leap Multi-Channel Psych Consortium, a broader multi-site program that seeks to develop integrated models of anhedonic depression.^1^

This sham-controlled, double-blinded study investigates the impact of multiple sessions of LIFUP targeting either the left caudate head or right nucleus accumbens on an array of anhedonia-related self-report and neuroimaging measures. The two targets for this study were selected due to the literature implicating them in the pathophysiology of anhedonic depression and their central role in mesolimbic reward circuitry (Haber and Knutson, 2010; Wang et al., 2021). Reduced caudate volume has been found to correlate with anhedonia severity, and both the nucleus accumbens and the caudate have been found to have significantly weaker reward responses in individuals with major depressive disorder when compared to healthy controls (Pizzagalli et al., 2009). The left caudate in particular was selected for this study due to literature suggesting that a longitudinal increase in left caudate connectivity with the bilateral superior frontal gyrus may correlate with anhedonia improvement over the same time period (Yang et al., 2022). The right nucleus accumbens was chosen based on literature showing a correlation between anhedonia severity and altered right nucleus accumbens connectivity (Liu et al., 2021). However, the right nucleus accumbens was removed from this study midway through due to funding changes.

A handful of studies have investigated the effect of DBS to the caudate on depressive symptoms. While the one study to-date of caudate DBS in individuals with major depression as their primary diagnosis found a lack of response, this study only tested caudate DBS in individuals for whom a similar-duration trial of nucleus accumbens DBS had already failed (Millet et al., 2014). In contrast to these findings, a study of caudate DBS in an individual with severe treatment resistant obsessive-compulsive disorder (OCD) and concomitant major depression observed remission of major depressive symptoms within 6 months of implantation, well-before remission of OCD symptoms was observed (Aouizerate et al., 2004). This suggests that the caudate may merit further investigation as a potential target for deep brain stimulation in depressive disorders.

DBS of the nucleus accumbens has also been found effective for depression, with one study finding a 50% response rate in participants with severely treatment-resistant depression in which an average of eight prior medical treatments had failed to sufficiently alleviate symptoms, including psychotherapy and electroconvulsive therapy (Bewernick et al., 2010). Additionally, nucleus accumbens DBS has been found to rapidly impact symptoms of anhedonia in depressed patients (Schlaepfer et al., 2008). Given this literature, the nucleus accumbens is a promising potential target for non-invasive brain stimulation with LIFUP.

Our study is the first to investigate the effects of LIFUP to the caudate and nucleus accumbens in individuals with anhedonic depression. However, preliminary studies in other populations have demonstrated the safety and feasibility of LIFUP to these regions. A recent study of LIFUP to the caudate in two veterans with post-traumatic headache observed reduced pain levels after several sessions (Yoon et al., 2024). Additionally, a study of long-term repeated administration of LIFUP to the caudate and putamen in non-human primates showed a slight but significant increase in motivation and task accuracy (Munoz et al., 2022). Stimulation of the nucleus accumbens using LIFUP has been found to reduce substance cravings in participants with substance use disorder and to increase connectivity between the nucleus accumbens and medial prefrontal cortex in healthy controls (Mahoney et al., 2023; Peng et al., 2024). Several other studies have also found beneficial effects of LIFUP to other brain regions in depressed participants, namely the subcallosal cingulate cortex and frontotemporal cortex (Reznik et al., 2020; Riis et al., 2024a,b).

Through this double-blinded, sham-controlled study, we hope to gain a better understanding of the feasibility and potential effects of LIFUP stimulation to the caudate head and nucleus accumbens in individuals with anhedonic depression. The study utilizes a wide array of self-report, digital phenotyping, and neuroimaging metrics in order to obtain a maximally comprehensive picture of the potential effects of LIFUP.

## 2 Materials and methods

### 2.1 Subject population

This protocol will enroll up to 100 right-handed adult participants with high anhedonia and moderate or high depression, with the goal of achieving an evaluable sample of at least 40 participants. For the purposes of this protocol, evaluable participants are defined as those who complete all three LIFUP sessions, both MRI scans, and at least one outcome measure after completing LIFUP.

#### 2.1.1 Inclusion/exclusion criteria

A complete list of inclusion/exclusion criteria for this study is available in Table 1.

**Table 1:** Inclusion and exclusion criteria.

**Inclusion criteria**	**Exclusion criteria**
Age 18–65	Current tobacco smoker of >11 cigarettes/day or the nicotine equivalent
Fluent in English	Current report of alcohol or substance abuse or dependence
Right-handed	Recent changes in antidepressant dosing or medication (dose and medication need to be stabilized within the last 2 weeks)
Healthy or corrected to healthy vision	Reported diagnosis of schizophrenia or psychotic symptoms
Residing in Los Angeles area for the duration of study	Currently taking benzodiazepines, or taken benzodiazepines in the past 8 weeks
Own functioning iOS smartphone (iPhone 8 or later, iOS 15 or newer) with access to reliable data plan and Wi-Fi	History of medical event(s) or diagnoses likely to result in neurological abnormalities including diagnoses of Alzheimer's, Parkinson's, neurodegenerative disorders, movement disorders, reports of past seizures or stroke, or history of brain tumors or brain surgery.
Able to read and understand a written informed consent form	Medical conditions involving chronic mobility impairment including spinal cord injuries, or severe osteoarthritis of knee or hip
Willing to participate in the study and complete required tasks as outlined in study timeline, including MRI, LIFUP, Apple Watch wear, and surveys	Unwilling or unable to refrain from making significant changes to hairstyle after enrollment and before LIFUP sessions are complete (i.e., full hairstyle to shaved head, significant change in locs, etc.)
Eligible for MRI scanning and neuromodulation	Contraindications for MRI scanning, including pregnancy, metal implants, braces, significant grip impairment and claustrophobia
PVSS < 6.5 at screening and stable for ≥12 weeks, as indicated by average scores across OPTIMA protocol or by self-report for those screened from general population	Any previous treatment with electroconvulsive therapy (ECT) or deep brain stimulation (DBS) due to increased risk of seizure and unclear evidence of how LIFUP will affect individuals who have received these treatments.
PHQ scores > 10 at screening and stable for ≥12 weeks, as indicated by average scores across OPTIMA protocol or by self-report for those screened from general population	Less than 6 months since any other neuromodulation treatment such as transcranial magnetic stimulation (TMS), Vagal nerve electrostimulation, or transcranial direct current stimulation (TDCS).
If screened from OPTIMA, have completed the majority of OPTIMA assessments	Less than 6 months since prescribed ketamine infusion or other intensive, acute therapy for depressive symptoms.

#### 2.1.2 Recruitment methods

A primary recruitment source for this study is the UCLA OPTIMA study. Study staff screen participants from OPTIMA who have consented for future contact for research and appear eligible based on their self-reported depression and anhedonia scores. The study is also recruiting individuals from the Los Angeles area based on self-reported depression and anhedonia scores.

#### 2.1.3 Participant retention

In order to promote participant retention, reminders for visits are sent throughout the study via e-mail, text message or phone call. Additionally, participants receive a final reminder about their in-person visits 24 h before each scheduled visit.

## 3 Data collection and intervention

**Figure 1:**
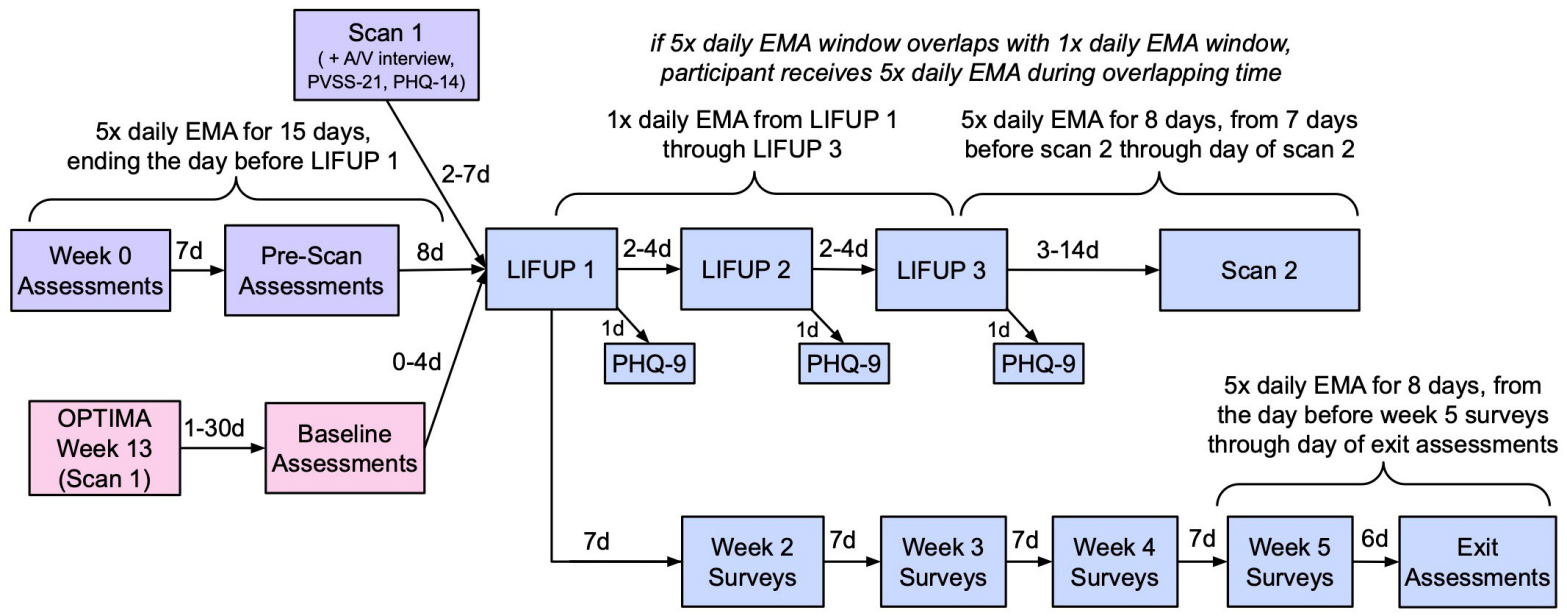
Study timeline. **Pink (bottom left)**: OPTIMA participants only. **Purple (top left)**: Non-OPTIMA participants only. **Blue (right)**: all participants.

For an overview of the timeline of data collection and intervention procedures described below, please refer to Figure 1.

### 3.1 Intervention protocol

#### 3.1.1 Condition assignment

Following screening and enrollment, participants are block-randomized to one of two conditions at equal rates: (1) active stimulation to the left caudate or (2) sham stimulation to the left caudate. A previous version of this protocol block-randomized participants into three conditions at equal rates: (1) active stimulation to the left caudate head, (2) active stimulation to the right nucleus accumbens, or (3) sham (assigned to left caudate or right nucleus accumbens to maintain concealment of conditions); however, due to budget changes, randomization to the “active stimulation to the right nucleus accumbens” condition was removed; a total of six participants were assigned to the active nucleus accumbens condition prior to its removal.

#### 3.1.2 LIFUP intervention

Participants receive the appropriate LIFUP intervention at each of three sessions; each session is spaced 2–4 days apart from the last, such that sessions occur at a minimum schedule of days 1, 3, 5 and at a maximum schedule of days 1, 5, 9. At each of these sessions, LIFUP is administered in ten 30-s sonications, with 30 s gaps between each sonication. LIFUP parameters are 5 ms pulse width, 100 Hz pulse repetition frequency, 50% duty cycle, ISPTA.3 720 mW/cm^2^, ISPPA.3 14.4 W/cm^2^. These parameters were selected due to previous literature suggesting that a 50% duty cycle can achieve excitatory effects (Zhang et al., 2021). The transducer is aimed at the appropriate target using BrainSight neuronavigation (BrainSight, Rogue Research, Montreal, Quebec, Canada) with a participant's T1-weighted structural scan. LIFUP is administered using the Brainsonix BX Pulsar 1002 focused ultrasound device with a 55, 65, or 80 mm transducer, depending on what depth is needed to reach the target for a given participant based on individual anatomy (Schafer et al., 2021). To minimize ultrasound energy exposure to air, a gel pad and water-based ultrasound gel are placed between the transducer and the participant's scalp. For active sonication, the transducer is used with a gel pad that allows the ultrasound energy to pass through; for sham sonication, the transducer is used with a gel pad that blocks ultrasound energy. Participants are asked to sit comfortably and remain still for the duration of the 10-min sonication procedure. If a participant expresses discomfort at any point, they are given the option to stop the procedure.

#### 3.1.3 Randomization and blinding

Active and sham conditions are concealed through the use of a set of identical gel pads factory-designated solely as A or B. The active and sham gel pads are visually identical to maintain blinding, and the retailer providing the gel pads (BrainSonix Corp., Sherman Oaks, California, United States) will maintain condition concealment until after the conclusion of the study. Staff are only informed which gel pad to use with blinded labels (A or B) and conduct all appointment procedures the same regardless of condition. As a result, staff performing the intervention, study investigators, and study participants all remain blinded to condition assignment. Participants are never informed if they received active or sham interventions. Condition assignments may be selectively revealed to research investigators and staff by steering committee consensus, when data analysis or research operations and monitoring require that conditions be known.

Randomization is performed via computer-generated random sequencing with blocking to reduce cohort effects. In the REDCap database, research staff are provided with a “randomize” function within each record, which provides them with condition assignment (i.e., gel pad A or B) after the command is executed.

The initial design of this study utilized three gel pads, one of which was sham. Participants in both the nucleus accumbens and caudate group were randomized to gel pads A, B, and C at equal rates, such that 1/3 of participants would receive active caudate, 1/3 would receive active nucleus accumbens, 1/6 would receive sham caudate, and 1/6 would receive sham nucleus accumbens. When it was determined due to funding changes that the study design should be reduced to two conditions, one individual on the team was unblinded by steering committee consensus. This individual then performed analyses on the imaging data from the Card Guessing Task to determine changes in reward attainment and anticipation across LIFUP in each group in order to guide a data-informed decision on which condition to keep in the study. Results of the analyses were then presented to the investigative team, keeping conditions concealed. Based on these results, investigators chose a condition to keep in the revised protocol, and an alternate condition was designated to keep in the case that the primary choice was sham. Investigators were informed that the nucleus accumbens target would be removed, but were not informed which group in the presented analyses was the active caudate group. The only individuals who were knowledgeable about condition assignment were the individual who performed the analysis and the research program manager who supervised the staff performing LIFUP. Conditions remained concealed to all other members of the research team. A total of six participants received active nucleus accumbens LIFUP prior to the removal of this condition.

To maximize the integrity of blinding, outcome assessors refrain from making any guesses regarding active/sham conditions. In the case where a participant guesses their condition based on how they feel during sessions, staff listen to their thoughts but refrain from discussion, and no records of these guesses are kept.

### 3.2 Self-report assessments

#### 3.2.1 Primary outcome assessments

The primary self-report outcome measures for this study are the Patient Health Questionnaire (PHQ-14), the Positive Valence Systems Scale (PVSS-21), and the Ecological Momentary Assessment (EMA). The PHQ-14 and PVSS-21 are completed weekly throughout the course of study participation; the schedule of EMA administration is described below and in Figure 1.

##### 3.2.1.1 Patient Health Questionnaire - 14 item version (PHQ-14)

The PHQ-14 is a modified version of the Patient Health Questionnaire (Kroenke et al., 2010) developed by Cohen, Cohen & Fried (in preparation)^2^ that separates the four compound symptoms from the original PHQ into individual items (e.g., “poor appetite or overeating” was split into two items) and adds two new symptoms: irritability and libido. Score is summarized as a total score aligned with the PHQ-8, where a higher score indicates the severity of depressive symptoms; further information and a copy of the full inventory are available in the Supplementary Method S4 and Supplementary Forms. This assessment was chosen as a primary outcome measure in order to provide information about any changes in overall depressive symptoms in response to LIFUP, given the association between depression symptoms and the LIFUP target regions.

##### 3.2.1.2 Positive Valence Systems Scale (PVSS-21)

The PVSS-21 (Khazanov et al., 2020) is a 21-item self-report survey assessing features of reward processing associated with anhedonia. This assessment was selected as a primary outcome measure due to the literature demonstrating connections between alterations in caudate and nucleus accumbens activity and anhedonia symptoms (Wang et al., 2021).

##### 3.2.1.3 Ecological Momentary Assessment (EMA)

The EMA is a 3-part, 19-item assessment that measures current mood and experiences.^3^ Part 1 contains 8 questions about current mood, e.g. “How sad are you feeling right now?” Part 2 asks participants to rate their agreement with 6 reward-related statements, such as “I'm looking forward to an upcoming activity”. Part 3 asks participants to rate their enjoyment of their current company and activities on a 7-point likert scale. This assessment was selected as a primary outcome measure due to its ability to provide data on immediate mood and reward-related states with high temporal resolution, thereby minimizing the impact of recall biases that have been documented for surveys asking about depressive symptoms over a longer time range (Horwitz et al., 2023).

For non-OPTIMA participants, baseline EMA data is collected 5 × a day for the 15 days prior to the first LIFUP session; for OPTIMA participants, baseline data is taken from the 5 × daily EMAs collected at several points during the OPTIMA protocol. All participants subsequently complete 1 × daily EMA from the day of LIFUP 1 through the day of LIFUP 3, and 8 days of 5 × daily EMAs starting 7 days prior to scan 2 through the day of scan. If scan 2 is < 8 days after LIFUP 3, the 5 × daily EMAs will override the 1 × daily EMAs on any overlapping days. Lastly, an additional 8 days of 5 × daily EMA data is collected during Week 5 of the protocol. See Figure 1 for an illustration of the EMA timeline. Further information regarding the development of this assessment and a copy of the full assessment are available in the Supplementary Method S1 and Supplementary Forms.

#### 3.2.2 Secondary outcome measures

An additional set of behavioral questionnaires assessing aspects of anhedonia, depression, anxiety, and general mental wellbeing are collected at baseline and at the end of the study. A list of assessments used for this study is available in Table 2, alongside descriptions of each assessment. Two secondary outcome assessments, The Modified SITBI-R + CSSRS-SR (MSC-SR)^4^ and the Eudaimonic Wellbeing Questionnaire,^5^ were developed by the study team for the purposes of this study; further information regarding the development of these assessments is available in the Supplementary Methods S2, S3. The schedule of administration for these measures is available in Supplementary Table S2, and full assessments are available in the Supplementary Forms.

**Table 2:** A list of the self-report questionnaires, neurocognitive tests, and interviews administered as part of the ILIAD protocol and descriptions of each measure.

**Assessment**	**Assessment type**	**Description**
Ecological Momentary Assessment (EMA)	Primary outcome	Self-reported current mood, environment, experiences, and anhedonic state
Positive Valence Systems Scale (PVSS-21)	Primary outcome	Self-reported index of anhedonia and reward processing, 21 items
Patient Health Questionnaire - modified (PHQ-14)	Primary outcome	Self-reported depression severity, 14 items
Patient Health Questionnaire (PHQ-9)	Secondary outcome/adverse event monitoring	Self-reported depression severity, 9 items
Quick Inventory of Depressive Symptoms (QIDS-SR15)	Secondary outcome	Self-reported depression symptoms, 15-items
Brief Irritability Test (BITe)	Secondary outcome	Self-report irritability measure, 5-items
Modified SITBI-R+CSSRS-SR (MSC-SR)	Secondary outcome	Self-report of suicidal ideation and behavior
Generalized Anxiety Disorder Questionnaire (GAD-7)	Secondary Outcome	Self-reported anxiety symptoms, 7-items
Work and Social Adjustment Scale (WSAS)	Secondary outcome	Self-reported functional impairment of current symptoms in work/school, social life, and family life
World Health Organization (WHO)-5 Wellbeing Index	Secondary outcome	Self-reported current mental wellbeing
Pittsburgh Sleep Quality Index (PSQI)	Secondary outcome	Self-reported recent sleep quality
Snaith-Hamilton Pleasure Scale (SHAPS)	Secondary outcome	Self-reported hedonic experience or positive valence
Apathy Motivation Index (AMI)	Secondary outcome	Self-report index of apathy and motivation
Ruminative Response Scale (RRS) brooding subscale (state)	Secondary outcome	Self-reported symptoms of rumination
Eudaimonic Wellbeing Questionnaire (EWBQ)	Secondary outcome	Self-reported wellbeing focused on values, meaning and purpose in life.
Treatment History Questionnaire	Covariate	Self-reported mental health treatment history (current/recent psychotherapy or psychiatric medication use)
Holmes Rahe Life Stress Survey (HRLSS)	Covariate	Self-reported exposure to moderate-to-major stressful life events
Prescribed Medication Questionnaire	Covariate	Self-report measure of currently prescribed medications, with a focus on psychiatric medications
Demographics Questionnaire	Covariate	Standard self-report measures of demographics (e.g., age, sex, ethnicity, education, etc.)
USDA Housing/Food Insecurity	Covariate	Self-report measure of household food security
Comorbidities Questionnaire	Covariate	Self-reported medical comorbidities commonly occurring with major depressive disorder
Trauma History Questionnaire	Covariate	Self-report measure of traumatic life events
Screening Assessment for Guiding Evaluation - Self-Report (SAGE-SR)	Covariate	An online, self-report-based comprehensive behavioral health diagnostic tool developed to cover the Diagnostic Statistical Manual (DSM); mirrors the Structured Clinical Interview for DSM Disorders.
TestMyBrain Neurocognitive battery (TMB)	Covariate	Computerized neurocognitive assessment; see Section 3.6
A/V recorded affect interview	Covariate	Seven-question affect induction interview; see Section 3.5.
Routines	Descriptive Measure	Self-report of daily activities and phone usage

Items listed as secondary outcome measures may also be utilized as covariates in some analyses. Full assessments and schedule of administration are available in the Supplementary material.

#### 3.2.3 Additional measures

In addition to primary and secondary outcome measures, participants will complete an array of assessments to obtain information about demographics and medical and psychiatric history. These will be used as covariates and sample descriptives, as well as potential outcome predictors in later analyses. These assessments are listed in Table 2 alongside descriptions of each metric.

### 3.3 Magnetic resonance imaging

All magnetic resonance imaging (MRI) data is obtained on a Siemens MAGNETOM Prisma 3 Tesla MRI scanner with a 32 channel head coil at the UCLA Staglin Center for Cognitive Neuroscience or the UCLA Ahmanson-Lovelace Brain Mapping Center. Prior to the MRI scan, all participants complete a comprehensive MRI safety screening to ensure their safety to undergo MRI. Participants are asked to remain still for the entire duration of the scan and are offered a short break halfway through the scan if needed. Participants recruited from the OPTIMA study receive their baseline MRI during week 13 of the OPTIMA protocol; the first LIFUP session typically is scheduled within 30 days of this scan. Participants who do not have MRIs from OPTIMA receive baseline scans 2–7 days prior to their first LIFUP session. All participants receive a second MRI scan 3–14 days after their third LIFUP session. All scans listed below are collected both at baseline and post-LIFUP, with the exception of the T2-weighted structural scan, which is only collected at baseline.

#### 3.3.1 Structural MRI

Two structural images are collected for each participant: a multi-echo T1-weighted structural image with 0.8 mm isotropic voxels, TR 2,500 ms, TEs 1.81 ms, 3.60 ms, 5.39 ms, 7.18 ms, TI 1,000 ms, FOV 256 mm, flip angle 8 degrees, GRAPPA acceleration factor 2, and a T2-weighted structural image with 0.8 mm isotropic voxels, TR 3,200 ms, TE 564 ms, FOV 256 mm, and GRAPPA acceleration factor 2.

#### 3.3.2 Resting state functional MRI

A total of 18 min of resting state fMRI data is collected, divided into two ~9-min, 300-volume scans with opposite phase encoding (anterior-posterior and posterior-anterior) but otherwise identical sequence parameters. Resting state fMRI is collected with a multi-echo, multi-band BOLD sequence with 2.5 mm isotropic voxels, TR 1,670 ms, TEs 15.60 ms, 38.20 ms, 60.80 ms, 83.40 ms, GRAPPA acceleration factor 2, multiband factor 4, echo spacing 0.50 ms. During the resting state fMRI scan, participants are asked to remain awake, look at a white crosshair presented on a black screen, and to think about nothing in particular. Active noise cancellation during resting state is done using OptoAcoustic noise canceling headphones (Optoacoustics Ltd, Mazor Israel).

#### 3.3.3 Functional MRI tasks

Two reward-based fMRI tasks are utilized for this study: the Apple Gathering Task and Card Guessing Task. For both tasks, fMRI is collected using a multi-band BOLD sequence with 2.0 mm isotropic voxels, TR 1,300 ms, TE 22.80 ms, multiband factor 3, FOV 208 mm, flip angle 64 degrees, echo spacing 0.53 degrees, GRAPPA acceleration factor 2. For each task, an additional brief (< 1 min) scan is collected prior to the task scan with identical sequence parameters but with opposite phase encoding. Active noise cancellation during functional MRI tasks is done using OptoAcoustic noise canceling headphones (Optoacoustics Ltd, Mazor Israel).

##### 3.3.3.1 Apple Gathering Task

The Apple Gathering Task (AGT) (Bonnelle et al., 2016; Armbruster-Genç et al., 2022) is an effort- and reward-based decision-making task. Participants are informed that they will receive an extra monetary reward based on their performance on this task; this reward ranges from $12 to $24. Participants are presented with a screen showing an apple tree with a variable number of apples (3, 6, 9, or 12). The apple tree is accompanied by a horizontal line across the tree trunk; the height of this line indicates the amount of effort that would be required to collect the apples on that tree (low, med-low, med-high, high), calibrated to each participant's personal maximum hand grip strength for each hand. Effort is expended by participants squeezing a hand-grip measurement tool (i.e., two dynamometers from Biopac Systems, Inc. using the MP160 and two DA100C amplifiers); this sends a signal to the screen, which then shows a vertical bar indicating the amount of effort they are expending. If participants successfully squeeze the hand grip above the required effort threshold for at least 2 s, they win all of the apples on the tree. If they do not successfully squeeze the hand grip, they do not win any apples that round. At the beginning of each trial, an apple tree is presented with a quantity of apples and required effort level; participants are then asked yes/no if they want to accept the trial or decline it. Upon acceptance of most trials, the apple tree will appear on either the left or the right side of the screen (counterbalanced), and participants will squeeze the grip measurement tool on the side where the tree appears to try to make their effort bar reach the horizontal line. In 25% of trials that are accepted, the participant does not need to squeeze the hand grip and instead simply acquires the apples. If the participant declines a trial, they move on to the next trial in which a new number of apples and amount of effort are offered. The task involves 80 trials in a 4 (Apples: 3, 6, 9, 12) × 4 (Effort: low, med-low, med-high, high) design with 5 trials for each of the 16 levels; each trial lasts 12 s. This task lasts up to 26 min in total, depending on the number of trials that the participant accepts. The AGT was originally administered during fMRI, but is now being collected as an outside-scanner task due to funding changes. Participants are informed of the monetary reward amount they will receive at the end of the scan (or at the end of the task, for those completing it outside of the scanner). The behavioral dependent variable is the participant's propensity to accept trials based on the number of apples and amount of effort. The primary neural dependent variables are reward anticipation (i.e., neural response when thinking about whether to accept or decline a trial) and response to reward attainment (i.e., neural response upon successfully obtaining apples).

##### 3.3.3.2 Card Guessing Task

The Card Guessing Task (CGT) (Forbes et al., 2009) is a reward-based task in which participants are informed that they will receive monetary reward for their performance on the task; however, the task is designed such that participants will have the same number of wins, losses, and neutral outcomes regardless of the choices they make, resulting in each participant receiving the same monetary reward. Participants are debriefed on this design at the end of their study participation. Prior to beginning the task, participants are told that the compensation will be as follows: +$6 for each win, -$2.50 for each loss, and no addition or deduction for neutral outcomes. Participants first have 3 s to guess whether the next randomly chosen card will be higher or lower than 5. If they do not guess within 3 s, the task skips to the next trial. Once a guess is submitted, it is briefly presented on the screen. Then, the participant is shown a screen revealing trial type; the three trial types are gain (win if correct, neutral if incorrect), loss (loss if incorrect, neutral if correct), and gain/loss (win if correct, loss if incorrect). Then, some number from 1 to 9 (excluding 5) is presented on the screen; this number is not fully random, but rather randomized within whatever range would align with the predetermined outcome for that trial based on the participant's guess. For example, if the predetermined outcome was for the guess to be correct, and the participant selected “high”, the screen would present some number from 6 to 9. Finally, the predetermined outcome feedback is presented, informing the participant if the outcome was a win (green up arrow and dollar sign to indicate gaining reward), loss (red down arrow and dollar sign with an “X” through it to indicate losing reward), or neutral (a yellow circle). The duration of each screen varies from trial to trial; the average trial duration across the task is 13 s. There are 36 trials, for a total task duration of ~8 min. There are no reward-based behavioral dependent variables in this task, as the only behavioral measure is the percentage of trials participants guess on, in which a low percentage would indicate inattention to the task. The main neural dependent variables are reward/loss anticipation and attainment of gains, losses, or neutral outcomes.

#### 3.3.4 Magnetic resonance spectroscopy

**Figure 2:**
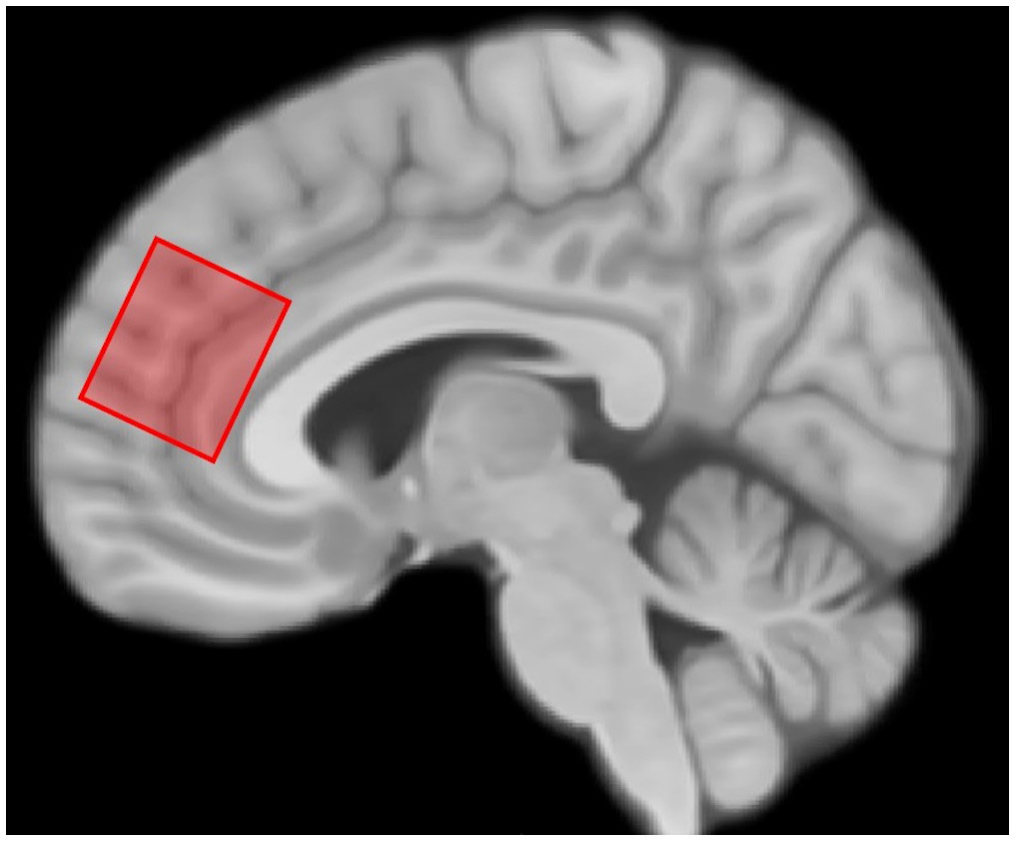
MRS voxel placement. The typical location and orientation of the MRS voxel overlaid on the MNI152 brain template.

Proton magnetic resonance spectroscopy (MRS) data is acquired using the HERMES sequence (TR: 2,000 ms; TE: 80 ms; 320 averages) (Chan et al., 2016; Saleh et al., 2016). This includes a full spectrum of brain metabolites as well as edited spectra for gamma-aminobutyric acid and glutathione quantification. A 2.5 × 2.5 × 3 cm voxel is placed on the midline of the brain in the prefrontal cortex and rotated to cover the dorsal anterior cingulate cortex and the dorsomedial prefrontal cortex (Figure 2). Siemens automatic shimming is used, with manual shimming if necessary, to achieve a displayed full-width half-max value of < 17 Hz for the unsuppressed water peak. Previous literature has demonstrated that at least in some regions of the brain, LIFUP is capable of altering metabolites as measured by MRS, though that study investigated the regions being stimulated rather than downstream regions (Yaakub et al., 2023). The placement of this voxel was chosen given literature suggesting alterations in an array of metabolites in the prefrontal cortex and anterior cingulate cortex of patients with depression (Xie et al., 2023). We anticipate that LIFUP to the caudate and nucleus accumbens may be able to alter metabolites in these regions due to the well-documented connections between the striatum and the anterior cingulate and medial prefrontal cortices as part of reward processing pathways (Haber, 2011).

#### 3.3.5 Field maps

For each functional MRI sequence, a pair of spin echo-based EPI images is collected with opposite phase encoding to each other, but matched along the axis of the phase encoding for the functional data and with identical voxel size to the corresponding sequence; this pair of images can be processed to create field maps to use for fMRI distortion correction.

### 3.4 Digital phenotyping

Participants in this study will wear an Apple Watch for the duration of the study in order to collect data on sleep, physical activity, heart rate, heart rate variability, and other digital measures available through Apple HealthKit and SensorKit. Participants will also download an app to their personal iPhone which collects additional digital measures, such as activity, environmental light, and wake and sleep patterns. A full list of data collected is available in Supplementary Tables S3, S4. This data is collected throughout the entirety of the 5 week protocol (7 weeks for non-OPTIMA participants). Participants continuing from OPTIMA are asked to continue wearing their Apple Watch during any gap between the end of the OPTIMA protocol and the beginning of the ILIAD protocol (typically < 30 days), such that these participants will have digital phenotyping data available for the same pre-LIFUP period as non-OPTIMA participants.

### 3.5 Affect interview

Participants complete an affect interview prior to the first MRI scan. This is a video-recorded interview in which an experimenter asks seven questions:

1. How are you feeling today?
2. Did anything happen this week that made you feel angry, stressed, or sad? If yes, describe what happened and how you felt.
3. Did anything happen this week that made you feel good? If yes, describe what happened and how you felt.
4. Is there something you are worrying about? If yes, please describe what is it you're worrying about and how it makes you feel.
5. Is there something good you expect will happen soon that you are looking forward to? If yes, what is it, and how does it make you feel?
6. Have you been planning or working toward a positive future event or experience? If yes, what is it and what have you been doing?
7. Tell me about your happiest memory.

Using sentiment analysis from natural language processing (bidirectional encoder representations from transformers; BERT) (Acheampong et al., 2021) of interview data, we will estimate the probability that the response to each interview question was positive, negative, or neutral in emotional valence. Specifically, we will use the roBERTa-base model, which is trained on 60 million tweets of varying levels of positive and negative sentiment, to estimate the sentiment of each interview question response (Barbieri et al., 2020). Due to the established risks of bias in natural language models trained on social media data, we aim to develop procedures to address potential model bias prior to final analysis of this data (Straw and Callison-Burch, 2020).

### 3.6 TestMyBrain neurocognitive tasks

Neurocognitive tasks are administered remotely through the use of TestMyBrain, a digital platform for cognitive testing. Table 3 lists the name of each task administered, the domain(s) of measurement, and a brief description of each task. A total of seven neurocognitive tasks are administered during pre-scan week (7 days before first scan). At study exit, two neurocognitive tasks are repeated: Multiracial Emotion Identification and Gradual Onset Continuous Performance Test. Tasks can be completed on a participant's smartphone or home computer. All tasks are presented in the fixed order listed in Table 3.

**Table 3:** A description of tasks administered in the TestMyBrain neurocognitive battery.

**Task name**	**Domain**	**Description**
Vocabulary (4 min)	Long-term verbal memory, crystallized cognitive ability, verbal reasoning	A vocabulary word is presented and the user must select 1 of 5 presented words that most closely matches the meaning of the vocabulary word.
Matrix reasoning (7.5 min)	Fluid cognitive ability, nonverbal reasoning	A series of image patterns are presented and the user is asked to select from options, which image will complete the pattern presented.
Multiracial emotion identification (3.5 min)	Face emotion perception, emotion identification	Photographs are presented of individuals expressing either anger, happiness, fear or sadness. The user must select which of the four emotions best describes the face presented.
Choice reaction item (1.5 min)	Processing speed, response selection/inhibition, attention	Three colored boxes are presented, each box contains an arrow that points left or right. Two boxes are the same color, one box is a different color. The user must indicate the direction of the arrow in the box that is a different color than the rest.
Digit symbol matching (2 min)	Processing Speed, visual short term memory	A key that matches numbers with symbols is shown, and the user is asked to match numbers to symbols as they are presented.
Multiple object tracking (5 min)	Visual working memory and visuospatial attention.	A set of target circles moves around the screen, among a larger set of identical distractor circles. The user must remember which circles were designated as targets, and track them throughout their movement alongside distractor circles.
Gradual onset continuous performance test (6 min)	Sustained attention, response inhibition, cognitive control	The user is presented with either a city image or a mountain image at varying intervals. For each image, the user must press a key when a city image appears and not press when a mountain image appears.

### 3.7 Data management and monitoring

#### 3.7.1 Data entry and storage

##### 3.7.1.1 Research Electronic Data Capture

This project utilizes REDCap for data collection and storage. REDCap employs relational database tables within a single MySQL database to store project records and all collected data. Survey data are collected directly from participants through External REDCap utilizing emailed survey links. Collected survey data are automatically synced from the external database to Internal REDCap. Non-survey data such as participant schedules, medication history, and records of in-person data collection are manually input by research staff and stored in Internal REDCap behind secure firewalls. Where third party services are utilized to collect survey data—as is the case for the SAGE Diagnostic Assessment and TestMyBrain Neurocognitive Assessment—External REDCap surveys are used to provide links to the third party site for assessment administration, and the REDCap API is utilized to copy that assessment data into the Internal REDCap database for secure storage within each record.

##### 3.7.1.2 The DGC Study App

The UCLA Depression Grand Challenge Study App (DGC Study App) built by Avicenna Research is installed on participant iPhones and used to collect digital health data. The DGC Study App uses HealthKit and SensorKit APIs for passive measures and deploys ecological momentary assessments (EMAs). The DGC Study App sends collected information to an Amazon Web Services (AWS) environment where EMA data and DGC Study App use activity are stored in a PostGreSQL server and passive sensor data (heart rate, accelerometer, etc.,) are stored in a CassandraDB instance. Data is periodically moved from the AWS server to a UCLA hosted Azure storage environment for storage and analysis.

#### 3.7.2 Data monitoring

Data quality is monitored weekly by the research staff and the program manager in order to ensure data collection occurred as prescribed by the protocol. Bi-weekly progress reports on participant compliance with digital phenotyping data collection are provided by the informatics team. Input and range restrictions for data entry are built into the database whenever possible. Research staff perform quality checks for all records of database entries that are transposed from paper records.

### 3.8 Trial management

#### 3.8.1 Trial registration

This study is registered on clinicaltrials.gov as NCT06285474. It has been approved by the UCLA Institutional Review Board as IRB#22-001323.

#### 3.8.2 Role of study sponsor and multi-institution collaboration

This study was developed by investigators at UCLA as part of the international and multi-institution Wellcome Leap Multi-Channel Psych Program (MCPsych). To facilitate harmonization of collection measures across the program and comparable outcome measures for joint analyses, sites synchronized collection of specific measures across several data collection modalities (i.e., self-report surveys, digital phenotyping, neuroimaging, etc.) in order to have harmonized outcome measures that would be comparable across sites in this consortium. Several protocol changes were initiated due to funding changes during the conduct of the study; the most notable of these changes were the removal of the nucleus accumbens LIFUP group (see Section 3.1) and the transition of the Apple Gathering Task to be outside of the scanner (see Section 3.3).

The trial sponsor is not responsible for any data collection, analysis, or interpretation related to this protocol. However, publications and presentations related to the data collected under the Wellcome Leap contract must receive approval from Wellcome Leap to ensure appropriate protection of intellectual property from other performing sites.

#### 3.8.3 Trial steering committee

The Trial Steering Committee for this protocol comprises the directors of the Depression Grand Challenge, principal and co-investigators of this protocol, as well as other professors, researchers, project scientists, and post-doctoral fellows. This group meets weekly with the research program manager to review study progress, data quality, deviations, protocol amendments, and participant care.

#### 3.8.4 Trial monitoring

A Data Monitoring Committee was not considered as this is a low-risk intervention. Protocol deviations are reported to the relevant centers as directed by the UCLA IRB and Office of Human Research Protection Program. Protocol amendments are communicated to investigative and research operations team members by the program manager through email notifications describing the changes and any impacts to operations. These email notifications include the updated protocol and relevant updated study documents. The clinical trial registry is kept up to date with protocol amendments and trial descriptions as changes are made.

#### 3.8.5 Research operations team

This study is being performed by research operations staff under the UCLA Depression Grand Challenge. The research operations project director provides supervision of study operations and ensures adherence to departmental standards. The project manager is responsible for protocol implementation, management of IRB submissions and study documents, database maintenance, data quality control, data monitoring, and study reporting. The research coordinator is responsible for participant screening, enrollment, and communication, as well as data collection and management. A team of research assessors is responsible for MRI data collection and managing participants in the MRI environment, as well as conducting LIFUP administration. Informatics specialists are an integral part of this protocol, as they are responsible for the management and storage of the multitude of data types, including digital sensing data, survey data, and scan data. Informatics specialists built the study screening website and are responsible for database technical support for this protocol. The study utilizes a third party company, Avicenna (previously Ethica) for app development and hosting to collect data for digital phenotyping and Ecological Momentary Assessment (EMA). This study collects information relating to suicidal ideation, and responses to participants that indicated imminent risk are managed in collaboration with Protocall Services—a third party, 24 h call service—as well as Depression Grand Challenge licensed clinical social workers.

#### 3.8.6 Adverse event monitoring

Adverse events are not explicitly solicited nor investigated under this protocol. However, monitoring for suicidality is conducted 24 h after each LIFUP appointment, as well as 72 h after the final LIFUP appointment. Spontaneously reported events are reported to the investigators in order to assess the probability that they were related to the study intervention. If events are determined to be possibly related, they are reported to the relevant centers as directed by the UCLA IRB and Office of Human Research Protection Program.

#### 3.8.7 Progress reports and auditing

Quarterly (once every 3 months) progress reports are provided to the study sponsor. These reports include data quality checks, interim analyses to evaluate protocol efficacy, and summary data. As a part of the larger program, our group participates in weekly communication with other sponsored sites as well as the trial sponsor in order to troubleshoot data collection methods and amend operating procedures as indicated. Auditing of trial conduct is performed by the investigators and the research operations team.

## 4 Planned analyses

### 4.1 Analysis of self-report measures

Primary analyses of self-report data will leverage multi-level modeling to assess the time course of response to LIFUP as well as the difference in response between the active and sham LIFUP groups. The active nucleus accumbens and active caudate conditions will be pooled for the primary analyses, with a second analysis being conducted with only the caudate group vs. sham. The primary variables of interest for these models will be total score on the PVSS and PHQ-14, and EMA variables measuring positive and negative affect. As a secondary analysis, we will re-run this multi-level modeling with the inclusion of the total score on the first eight items of the PHQ-9 (such that total score aligns with PHQ-14 total score) administered one day after each LIFUP session.

Additionally, chi-square analyses will be conducted to assess differences in response rate between sham and active LIFUP, where response will be defined as a 25% or greater score reduction on PHQ-14 or score increase on the PVSS between baseline and the Week 3 survey timepoint (i.e., ~1 week post-LIFUP). We hypothesize that active stimulation of either caudate or nucleus accumbens will be associated with a decrease in PHQ-14 score and increase in PVSS score relative to sham stimulation. This hypothesis is supported by literature showing associations between decreased reward sensitivity in anhedonic depression and hypoactivity in the striatal regions that this study aims to excite (Wang et al., 2021). Similarly, we hypothesize that the EMA data will show an increase in positive affect and a decrease in negative affect after active stimulation relative to sham.

For secondary outcome measures with only two timepoints, repeated measures ANOVA will be used to investigate the effect of time (pre vs. post-LIFUP) and group (active caudate vs. active nucleus accumbens vs. sham).

### 4.2 Analysis of MRI data

Primary imaging analyses will include pre vs. post-LIFUP analysis of reward processing, activation, and connectivity of the caudate and nucleus accumbens as measured with resting state fMRI and task fMRI. Preprocessing of functional MRI data will be done with fMRIprep, Tedana, and XCP-D (Esteban et al., 2020; DuPre et al., 2021; Mehta et al., 2023). Resting state and task fMRI analyses will be conducted using the fMRIB Software Library (FSL) (Woolrich et al., 2001, 2004).

The functional MRI data collected during the Card Guessing Task will be used to assess changes in reward processing across active and sham LIFUP. Reward processing will be assessed by comparing fMRI activation during gain vs. loss trials as well as during gain trials vs. inter-trial intervals. Then, a repeated-measures ANOVA design will be used to assess the effect of time and group on these lower-level contrasts. We hypothesize that participants who received active LIFUP will have higher reward-responsive activity in their respective target regions (i.e., either nucleus accumbens or caudate) compared to those who received sham. This would represent a normalization of activity, as decreased activity has been observed in both regions in response to reward-related tasks (Wang et al., 2021).

Connectivity values between multiple regions of interest will be extracted from the resting state fMRI data in order to investigate changes across LIFUP vs. sham using multi-level modeling. Some particular variables of interest for this analysis due to prior implications for anhedonia in the literature include default mode network average connectivity, left caudate to superior frontal gyrus connectivity, right nucleus accumbens shell to left subgenual anterior cingulate cortex, and right nucleus accumbens core to right precuneus (Dunlop et al., 2019; Liu et al., 2021; Yang et al., 2022).

Additionally, several metabolites measured by MRS will be investigated to elucidate potential changes in reward system neurotransmission across LIFUP vs. sham. MRS data will be processed using Osprey, a software platform developed by the same team that developed the HERMES MRS sequence used for this study (Oeltzschner et al., 2020). Osprey reports metabolite concentrations, relative to water, after correcting for voxel tissue composition and water relaxation rates. GABA+ measurements are additionally corrected to account for differing concentrations in gray and white matter. Primary variables of interest for analysis of the MRS data due to their associations with depression and anhedonia in the literature include concentrations of GABA, Glx (sum of glutamate and glutamine), and glutathione (Sanacora et al., 2012; Luscher and Fuchs, 2015; Tuura et al., 2023). We hypothesize that active LIFUP will result in increases in both GABA and Glx concentrations in the dorsomedial prefrontal cortex, representing a normalization in neurotransmitter levels similar to that observed after successful treatment with other modalities (Sanacora et al., 2003; Chen et al., 2014). Furthermore, we hypothesize that we will observe increased glutathione levels after active LIFUP relative to sham, given our hypothesis of decreased anhedonia and prior findings of lower glutathione in anhedonic participants (Lapidus et al., 2014).

### 4.3 Analysis of digital phenotyping data

Primary variables of interest from the digital phenotyping data include heart rate, heart rate variability, sleep duration, and active energy burned. Multi-level modeling will be utilized to investigate between-group differences in these measures across LIFUP (or sham) stimulation. After analysis of these primary variables, further exploratory analyses will be conducted with a broader set of digital phenotyping variables; selection of these variables will likely be guided by OPTIMA analyses assessing correlations between digital phenotyping measures and anhedonia severity.

### 4.4 Predictive analyses

Two primary sets of predictive analyses will be conducted utilizing a machine learning approach, both aiming to identify predictors of LIFUP response as measured by change in score on the PVSS and PHQ-14. First, in order to maximize power given the relatively small sample size of this study, a smaller subset of potential predictors (~50) will be analyzed. These predictors will be selected based on both the literature and the results of analyses from the OPTIMA study exploring potential MRI, digital phenotyping, and self-report-based predictors of anhedonia severity. Subsequently, an exploratory bottom-up approach will be used to investigate other potential predictors that were not in the initial subset. This analysis will utilize all features across all data modalities in conjunction with ensemble machine learning techniques including dimensionality reduction and automated machine learning.

### 4.5 *Post-hoc* exploratory analyses

Given the breadth of data collected through this protocol, a wide variety of both hypothesis-based and exploratory analyses will be possible that have not yet been developed.

## 5 Discussion

Direct stimulation of reward-related subcortical regions is an approach that has proven highly effective in individuals with major depressive disorder (Figee et al., 2022). However, due to the invasiveness and risks associated with surgical DBS, the studies conducted to date have been primarily open-label, small-sample studies of participants with severe, highly treatment-resistant depression. The investigation of caudate and nucleus accumbens stimulation with LIFUP in this protocol will therefore help to elucidate the potential efficacy of this intervention in individuals with a wider range of depression severity and treatment resistance than those who have been studied previously. Furthermore, despite the literature implicating the caudate in the mechanism of anhedonic depression, direct stimulation of the caudate has been minimally investigated as a potential treatment for depressive symptoms. If caudate stimulation is found to be effective through this protocol, it could highlight a new potential target for both invasive and non-invasive deep brain stimulation for individuals with depressive disorders.

This study is also one of the first studies to investigate the effects of multiple sessions of deep brain LIFUP on depressive and anhedonic symptoms; previous multi-session LIFUP studies in clinical populations have found significant impacts on anxiety symptoms (Mahdavi et al., 2023), autism spectrum disorder symptoms (Cheung et al., 2023), and worry symptoms (Reznik et al., 2020). Furthermore, a single, 40-min session of LIFUP to the subcallosal cingulate cortex was recently found to reduce depressive symptoms in individuals with treatment resistant depression (Riis et al., 2024a,b). However, none of these previous studies included longitudinal neuroimaging measures or continuous physiological monitoring. As a result, this study is a critical step forward in the investigation of the potential neurological and physiological effects of multi-session deep brain LIFUP. This is especially important given that most other non-invasive brain stimulation techniques used in psychiatric applications, such as transcranial magnetic stimulation (TMS) and transcranial direct current stimulation (TDCS) require multiple sessions for optimal treatment response (Li et al., 2022; Hutton et al., 2023). While this study still does not reach the 10+ sessions typically used in TMS and TDCS applications, a demonstration of the feasibility and safety of multi-session LIFUP through this protocol would provide support for the investigation of more intensive LIFUP protocols in future studies.

A notable limitation of this study protocol is the timeline variability across participants. The time course of LIFUP effects after multiple consecutive sessions has not yet been investigated and is therefore unknown. Thus, while the 3–14 day window between the last LIFUP session and the post-LIFUP MRI is necessary for the logistical feasibility of the study, there is a chance that the effects observed in the neuroimaging data for each participant may be strongly impacted or even rendered undetectable by the relative timing of the post-LIFUP MRI. This is a critical confound that will need to be controlled for in analyses; however, this range of timepoints may also facilitate investigation of the time course of post-LIFUP changes in neurological function.

Due to budget changes partway through the study, it was necessary to remove the nucleus accumbens condition from this study. As a result, the nucleus accumbens group sample size is markedly smaller than initially intended, limiting the power of subsequent analyses for this target. However, this reduced sample (six active participants) still represents the largest study to date of nucleus accumbens LIFUP, and the only one in depressed participants (Mahoney et al., 2023; Peng et al., 2024). Furthermore, the previous studies found significant effects even with smaller samples, suggesting that the data collected for this condition may still be sufficient to detect potential effects (Mahoney et al., 2023; Peng et al., 2024). Another sample-related limitation lies in the nature of study participation; depressed participants who are willing and able to participate in a research study requiring multiple in-person visits and an array of remote tasks may have different characteristics and symptom profiles than the broader population of individuals with anhedonic depression, limiting the generalizability of our data.

In conclusion, this double-blinded, sham-controlled study aims to investigate the effects of non-invasive subcortical brain stimulation with LIFUP to the left caudate head and right nucleus accumbens in individuals with anhedonic depression. Through the extensive MRI, self-report, and digital phenotyping data being collected through this protocol, it will be possible to investigate a wide variety of potential effects of LIFUP, as well as an array of potential predictors of response. If LIFUP is found to effectively modulate brain activity and levels of anhedonia, it could open up a path toward a new, critically-needed alternative to surgical deep brain stimulation in patients with treatment-resistant depression.

## Footnotes

^1^ https://wellcomeleap.org/mcpsych/

^2^see https://osf.io/w4rj9/ and Supplementary material for more information.

^3^EMA items developed in consultation with Eiko Fried, Gabi Khazanov, and Courtney Forbes, and drawn from the SCOUT study (Kirtley et al., 2024), WARN-D (Fried et al., 2023), and the ESM Item Repository (Kirtley et al., 2018).

^4^Lifetime and current suicidality measure that combines items from the CSSRS-SR (Posner et al., 2011) and the SITBI-R (Fox et al., 2020); adapted in consultation with Kathryn Fox and Shirley Wang.

^5^Adapted in consultation with Sandman (2023) from the Mental Health Continuum Short Form (MCH-SF; Keyes et al., 2008) and Daily Meaning Scale (DMS) (Steger et al., 2008).
