# Supplementary material for: Investigating low intensity focused ultrasound pulsation in anhedonic depression—A randomized controlled trial
Source: Front Hum Neurosci. 2025 Mar 24;19:1478534. doi: 10.3389/fnhum.2025.1478534 (PMC11973349; doi:10.3389/fnhum.2025.1478534)
Supplement: Supplementary file 1 [file Data_Sheet_1.pdf]

Supplementary Forms

Table of Contents

[2 - 3] Ecological Momentary Assessment (EMA)
[4] Patient Health Questionnaire (PHQ-9)
[5] Patient Health Questionnaire - modified (PHQ-14)
[6] Positive Valence Systems Scale (PVSS-21)
[7] Edinburgh Handedness Inventory
[8 - 9] MRI Screening Form
[10 - 30] Prescribed Medications
[31 - 35] Demographics Questionnaire
[36 - 39] Quick Inventory of Depressive Symptoms (QIDS)
[40] Brief Irritability Test (BITe)
[41 - 43] Modified SITBI-R + CSSRS-SR (MSC-SR)
[44 - 45] Generalized Anxiety Disorder Questionnaire (GAD-7)
[46] Work and Social Adjustment Scale (WSAS)
[47] WHO-5 Wellbeing Index
[48 - 51] Pittsburgh Sleep Quality Index (PSQI)
[52] Snaith-Hamilton Pleasure Scale (SHAPS)
[53 - 54] Apathy Motivation Index (AMI)
[55] RRS-brooding subscale
[56] Eudaimonic Well-Being Questionnaire (EWBQ)
[57 - 61] USDA Housing/ Food Insecurity Questionnaire
[62 - 63] Comorbidities Questionnaire
[64] Treatment History Questionnaire
[65 - 72] Routines Questionnaire
[73 - 75] Holmes Rahe Life Stress Survey
[76 - 78] Trauma History Questionnaire

	1<br>Not<br>at all	2<br>Slightly	3<br>Moderately	4<br>Very	5<br>Extremely
How sad do you feel right now	☐ 1	☐ 2	☐ 3	☐ 4	☐ 5
How stressed do you feel right now	☐ 1	☐ 2	☐ 3	☐ 4	☐ 5
How anxious do you feel right now	☐ 1	☐ 2	☐ 3	☐ 4	☐ 5
How annoyed/irritated do you feel right now	☐ 1	☐ 2	☐ 3	☐ 4	☐ 5
How energetic do you feel right now	☐ 1	☐ 2	☐ 3	☐ 4	☐ 5
How happy do you feel right now	☐ 1	☐ 2	☐ 3	☐ 4	☐ 5
How motivated do you feel right now	☐ 1	☐ 2	☐ 3	☐ 4	☐ 5
How engaged do you feel right now	☐ 1	☐ 2	☐ 3	☐ 4	☐ 5
How lonely do you feel right now	☐ 1	☐ 2	☐ 3	☐ 4	☐ 5

Right now... I'm looking forward to an upcoming activity	True<br>☐	False<br>☐
Right now...I'm feeling good after doing something	True<br>☐	False<br>☐
Right now… I'm putting effort into planning something that will be enjoyable in the future	True<br>☐	False<br>☐
Right now… I could be doing something positive but am not because I don't think I'd enjoy it	True<br>☐	False<br>☐
Right now… I could be doing something positive but am not because it feels like too much effort	True<br>☐	False<br>☐
Right now… I'm feeling a sense of meaning and purpose	True<br>☐	False<br>☐

1. Right now, I am with (includes phone/video/digital) choose all that apply:
   - ☐ Friend
   - ☐ Family
   - ☐ Romantic partner
   - ☐ Co-worker/classmate/acquaintance
   - ☐ Stranger
   - ☐ Pet
   - ☐ Other
   - ☐ No one

(skip if answer to #1 is "no one")

2. I am enjoying this company:

- ☐ 1: Not at all
- ☐ 2
- ☐ 3
- ☐ 4
- ☐ 5
- ☐ 6
- ☐ 7: Very much

3. Right now, I am doing (choose all that apply):

- ☐ Social activity (offline or online)
- ☐ Psychical activity (e.g. gym, sports)
- ☐ Active leisure activity (e.g. hobby, board game)
- ☐ Passive leisure activity (e.g. watching TV, scrolling Instagram)
- ☐ Working/studying
- ☐ Tasks (e.g. cleaning house)
- ☐ On my way to somewhere
- ☐ Eating
- ☐ Childcare
- ☐ Other
- ☐ nothing

4. I am enjoying what I am doing

- ☐ 1: Not at all
- ☐ 2
- ☐ 3
- ☐ 4
- ☐ 5
- ☐ 6
- ☐ 7: Very much

PATIENT HEALTH QUESTIONNAIRE-9 (PHQ-9)

**Over the last 2 weeks, how often have you been bothered by any of the following problems?** *(Use "✔" to indicate your answer)*	**Not at all**	**Several days**	**More than half the days**	**Nearly every day**
**1.** Little interest or pleasure in doing things	0	1	2	3
**2.** Feeling down, depressed, or hopeless	0	1	2	3
**3.** Trouble falling or staying asleep, or sleeping too much	0	1	2	3
**4.** Feeling tired or having little energy	0	1	2	3
**5.** Poor appetite or overeating	0	1	2	3
**6.** Feeling bad about yourself — or that you are a failure or have let yourself or your family down	0	1	2	3
**7.** Trouble concentrating on things, such as reading the newspaper or watching television	0	1	2	3
**8.** Moving or speaking so slowly that other people could have noticed? Or the opposite — being so fidgety or restless that you have been moving around a lot more than usual	0	1	2	3
**9.** Thoughts that you would be better off dead or of hurting yourself in some way	0	1	2	3

FOR OFFICE CODING ___0___ + ______ + ______ + ______

=Total Score: ______

If you checked off any problems, how difficult have these problems made it for you to do your work, take care of things at home, or get along with other people?

**Not difficult at all**	**Somewhat difficult**	**Very difficult**	**Extremely difficult**
☐	☐	☐	☐

Developed by Drs. Robert L. Spitzer, Janet B.W. Williams, Kurt Kroenke and colleagues, with an educational grant from Pfizer Inc. No permission required to reproduce, translate, display or distribute.

The Patient Health Questionnaire 1 week

**Over the *last week*, how often have you been bothered by the following problems?**	**Not At all**	**Several Days**	**More Than Half the Days**	**Nearly Every Day**
1. Little interest or pleasure in doing things	0	1	2	3
2. Feeling down or depressed	0	1	2	3
3. Feeling hopeless	0	1	2	3
4. Trouble falling asleep or staying asleep	0	1	2	3
5. Sleeping too much	0	1	2	3
6. Feeling tired or having little energy	0	1	2	3
7. Poor appetite	0	1	2	3
8. Overeating	0	1	2	3
9. Feeling bad about yourself – or that you're a failure or have let yourself or your family down	0	1	2	3
10. Trouble concentrating on things, such as reading or watching television	0	1	2	3
11. Moving or speaking so slowly that other people could have noticed	0	1	2	3
12. Being so fidgety or restless that you have been moving around a lot more than usual	0	1	2	3
13. Feeling irritable	0	1	2	3
14. Little interest in sex	0	1	2	3

Positive Valence Systems Scale, 21 items (PVSS-21)

Please indicate to what extent these statements describe your **responses over the last week, including today.**

Did you NOT have this experience? No problem. Please indicate how you would have responded if you had experienced the situation over the last two weeks.

Please consider only the aspect of the situation that is described, paying particular attention to the underlined text. For example, if the statement says, "I wanted to meet new people," rate how much you wanted or would have wanted to meet new people over the last week, assuming that the opportunity presented itself. Do not consider what the situation would have required of you or whether it would have been possible for you to meet people.

1------------2------------3------------4------------5------------6------------7------------8------------9

1	2	3	4	5	6	7	8	9
Extremely untrue of me	Very untrue of me	Moderately untrue of me	Slightly untrue of me	Neutral	Slightly true of me	Moderately true of me	Very true of me	Extremely true of me

1. I savored my first bite of food after feeling hungry
2. I put energy into activities I enjoy
3. I was delighted to catch a breath of fresh air outdoors
4. I wanted to spend time with people I know
5. A fun activity during the weekend sustained my good mood throughout the new week
6. It felt good to have physical contact with someone I felt close to
7. I expected to enjoy a brief moment outdoors
8. I looked forward to hearing feedback on my work
9. I expected to enjoy my meals
10. Receiving praise about my work made me feel pleased for the rest of the day
11. I looked forward to spending time with others
12. I wanted to accomplish goals I set for myself
13. I expected to enjoy being hugged by someone I love
14. I wanted to participate in a fun activity with friends
15. I worked hard to earn positive feedback on my projects
16. I looked forward to an upcoming meal
17. I felt pleased when I reached a goal I set for myself
18. Getting a hug from someone close to me made me happy even after we parted
19. I expected to master the tasks I undertook
20. I actively pursued activities I thought would be fun
21. I went out of my way to admire the beauty around me

Date: ___/___/___

Coded Study Number: ______________________________

Edinburgh Handedness Inventory (revised)					
Please mark the box that best describes which hand you use for the activity in question					
	Always Left	*Usually Left*	*No Preference*	*Usually Right*	*Always Right*
**Writing**					
**Throwing**					
**Scissors**					
**Toothbrush**					
**Knife (without fork)**					
**Spoon**					
**Match (when striking)**					
**Computer mouse**					

Always Left = 1

Usually Left = 2

No Preference = 3

Usually Right = 4

Always Right = 5

These numbers are used to do the calculation below.

Scoring:
After calculating the sums above for total number of right-handed, left-handed, and no preference responses, we insert those numbers into the formula below to give us the final score:

(Right - Left) / (Right + Left).

Staglin Center for Cognitive Neuroscience

MRI Screening Form

STAGLIN Center for Cognitive Neuroscience

First name: ______________________ Last name: ______________________ Date of birth: ____/____/______

Height: __________ Weight: __________ Investigator name or protocol: ______________________

WARNING: MRI is generally very safe. However, certain implants, devices, or objects may be hazardous to you and/or may interfere with the MR procedure (i.e., MRI, MR angiography, functional MRI, MR spectroscopy). Do not enter the MR system room or MR environment if you have any question or concern regarding an implant, device, or object. Consult the MRI Technologist or Investigator BEFORE entering the MR system room. The MR system magnet is ALWAYS on.

1. Have you had prior surgery or an operation (e.g., arthroscopy, endoscopy, etc.) of any kind, particularly in the last six weeks? .... ☐ Yes ☐ No

   If yes, please indicate the date and type of surgery:

   Type of surgery (list all, if more than one) ______________________

   ____

2. Have you experienced any problem related to a previous MRI examination or MR procedure? .... ☐ Yes ☐ No

   If yes, please describe: ______________________

3. Have you had an injury to the eye involving a metallic object or fragment (e.g., metallic slivers, shavings, foreign body, etc.)? .... ☐ Yes ☐ No

   If yes, please describe: ______________________

4. Have you ever been injured by a metallic object or foreign body (e.g., BB, bullet, shrapnel, etc.)? .... ☐ Yes ☐ No

   If yes, please describe: ______________________

5. Are you currently taking or have you recently taken any medication or drug? .... ☐ Yes ☐ No

   If yes, please list: ______________________

6. Do you have diabetes? .... ☐ Yes ☐ No

7. Do you have cardiac hypertension? .... ☐ Yes ☐ No

8. Do you take beta blockers? .... ☐ Yes ☐ No

9. Are you taking sedatives? .... ☐ Yes ☐ No

10. Do you take diuretics? .... ☐ Yes ☐ No

11. Do you have a fever? .... ☐ Yes ☐ No

For female subjects:

12. Are you pregnant or experiencing a late menstrual period? .... ☐ Yes ☐ No

13. Are you taking any type of fertility medication or having fertility treatments? .... ☐ Yes ☐ No

Staglin Center for Cognitive Neuroscience

MRI Screening Form

STAGLIN Center for Cognitive Neuroscience

Please indicate if you have any of the following:

☐ Yes	☐ No	Aneurysm clip(s)	☐ Yes	☐ No	Vascular access port and/or catheter
☐ Yes	☐ No	Cardiac pacemaker	☐ Yes	☐ No	Radiation seeds or implants
☐ Yes	☐ No	Implanted cardioverter defibrillator (ICD)	☐ Yes	☐ No	Swan-Ganz or thermodilution catheter
☐ Yes	☐ No	Electronic implant or device	☐ Yes	☐ No	Medication patch (Nicotine, Nitroglycerine)
☐ Yes	☐ No	Magnetically-activated implant or device	☐ Yes	☐ No	Any metallic fragment or foreign body
☐ Yes	☐ No	Neurostimulation system	☐ Yes	☐ No	Wire mesh implant
☐ Yes	☐ No	Spinal cord stimulator	☐ Yes	☐ No	Tissue expander (e.g., breast)
☐ Yes	☐ No	Internal electrodes or wires	☐ Yes	☐ No	Surgical staples, clips, or metallic sutures
☐ Yes	☐ No	Bone growth/bone fusion stimulator	☐ Yes	☐ No	Joint replacement (hip, knee, etc.)
☐ Yes	☐ No	Cochlear, otologic, or other ear implant	☐ Yes	☐ No	Bone/joint pin, screw, nail, wire, plate, etc.
☐ Yes	☐ No	Insulin or other infusion pump	☐ Yes	☐ No	IUD, diaphragm, or pessary
☐ Yes	☐ No	Implanted drug infusion device	☐ Yes	☐ No	Dentures or partial plates
☐ Yes	☐ No	Any type of prosthesis (eye, penile, etc.)	☐ Yes	☐ No	Tattoo or permanent makeup
☐ Yes	☐ No	Heart valve prosthesis	☐ Yes	☐ No	Body piercing jewelry
☐ Yes	☐ No	Eyelid spring or wire	☐ Yes	☐ No	Hearing aid
☐ Yes	☐ No	Artificial or prosthetic limb	☐ Yes	☐ No	Other implant ______________________
☐ Yes	☐ No	Metallic stent, filter, or coil	☐ Yes	☐ No	Breathing problem or motion disorder
☐ Yes	☐ No	Shunt (spinal or intraventricular)	☐ Yes	☐ No	Claustrophobia

Notes

____

____

____

____

____

____

____

Before entering the MR environment or MR system room, you must remove all metallic objects including hearing aids, dentures, partial plates, keys, beeper, cell phone, eyeglasses, hair pins, barrettes, jewelry, body piercing jewelry, watch, safety pins, paperclips, money clip, credit cards, bank cards, magnetic strip cards, coins, pens, pocket knife, nail clipper, tools, clothing with metal fasteners, & clothing with metallic threads.

Please consult the MRI Technologist or Investigator if you have any question or concern BEFORE you enter the MR system room.

I attest that the above information is correct to the best of my knowledge. I have read and understand the contents of this form and had the opportunity to ask questions regarding the information on this form and regarding the MR procedure that I am about to undergo.

Signature of Person Completing Form: ___________________________________ Date: _____/____/_______
Signature

Form Completed By: ☐ Subject ☐ Relative ☐ Other ____
Print name and Relationship to subject

Form Information Reviewed By: ____
Print name and Sign

☐ MRI Technologist ☐ Investigator ☐ Other: ____

Prescribed Medication History

Study ID ______________________

Today's date:
Please record start time ______________________

Are you allergic to any medications? ◯ Yes ◯ No

Please describe: ______________________

I'm going to ask you to share with me all current medications that you are taking. To make sure we capture everything, I will go through a number of categories, and offer a few examples within each category. If you are taking a medication in one of these categories, I will then ask you for more information about current dose, and duration of use.

At this time, I won't be asking you about medications that you have taken in the past - just medications, whether prescribed or not, that you are taking now.

For those participants that didn't come prepared with a list, or seem uncertain, you can list more medication names when reviewing each category above.

You can additionally ask "Are you certain that this is the full list of medications that you are currently taking? We want to capture everything that you may be taking."

Are you taking any antidepressant medications known as SSRIs?
SSRI medications include Celexa, Lexapro, Paxil, Prozac, and others.

	Current Use
Celexa (Citalopram)	◯
Lexapro (Escitalopram)	◯
Paxil (Paroxetine)	◯
Prozac (Fluoxetine)	◯
Zoloft (Sertraline)	◯
Other SSRI - Serzone (Nefazodone), Luvox (Fluvoxamine):	◯

Current Use: Celexa Dose ______________________

Current Use: Date (Month/Year) Celexa First Taken ______________________

Current Use: Date (Month/Day/Year) Celexa Last Taken ______________________

Duration (Celexa) - Months ______________________

Current Use: Lexapro Dose
____

Current Use: Date (Month/Year) Lexapro First Taken
____

Current Use: Date (Month/Day/Year) Lexapro Last Taken
____

Duration (Lexapro) - Months
____

Current Use: Paxil Dose
____

Current Use: Date (Month/Year) Paxil First Taken
____

Current Use: Date (Month/Day/Year) Paxil Last Taken
____

Duration (Paxil) - Months
____

Current Use: Prozac Dose
____

Current Use: Date (Month/Year) Prozac First Taken
____

Current Use: Date (Month/Day/Year) Prozac Last Taken
____

Duration (Prozac) - Months
____

Current Use: Zoloft Dose
____

Current Use: Date (Month/Year) Zoloft First Taken
____

Current Use: Date (Month/Day/Year) Zoloft Last Taken
____

Duration (Zoloft) - Months
____

Current Use: Other Dose
____

Current Use: Date (Month/Year) Other First Taken
____

Current Use: Date (Month/Day/Year) Other Last Taken

Duration (Other) - Months

Are you taking any antidepressant or anti-anxiety medications known as SNRIs?
SNRI medications include Effexor, Cymbalta, and others.

	Current Use
Effexor (Venlafaxine)	○
Pristiq (Desvenlafaxine)	○
Savella (Milnacipran)	○
Cymbalta (Duloxetine)	○
Fetzima (Levomilnacipran)	○
Irenka (Duloxetine)	○
Khedezla (Desvenlafaxine)	○

Current Use: Effexor Dose

Current Use: Date (Month/Year) Effexor First Taken

Current Use: Date (Month/Day/Year) Effexor Last Taken

Duration (Effexor) - Months

Current Use: Pristiq Dose

Current Use: Date (Month/Year) Pristiq First Taken

Current Use: Date (Month/Day/Year) Pristiq Last Taken

Duration (Pristiq) - Months

Current Use: Savella Dose

Current Use: Date (Month/Year) Savella First Taken

Current Use: Date (Month/Day/Year) Savella Last Taken

Duration (Savella) - Months

Current Use: Cymbalta Dose

____

Current Use: Date (Month/Year) Cymbalta First Taken

____

Current Use: Date (Month/Day/Year) Cymbalta Last Taken

____

Duration (Cymbalta) - Months

____

Current Use: Fetzima Dose

____

Current Use: Date (Month/Year) Fetzima First Taken

____

Current Use: Date (Month/Day/Year) Fetzima Last Taken

____

Duration (Fetzima) - Months

____

Current Use: Irenka Dose

____

Current Use: Date (Month/Year) Irenka First Taken

____

Current Use: Date (Month/Day/Year) Irenka Last Taken

____

Duration (Irenka) - Months

____

Current Use: Khedezla Dose

____

Current Use: Date (Month/Year) Khedezla First Taken

____

Current Use: Date (Month/Day/Year) Khedezla Last Taken

____

Duration (Khedezla) - Months

____

Are you taking any other antidepressant medications, such as Wellbutrin or Nardil?

	Current Use
Anafranil (Clominpramine)	○
Desyrel (Trazadone)	○
Nardil (Phenelzine)	○
Norpramine (Desipramine)	○
Parnate (Tranylcypromine)	○
Remeron (Mirtazapine)	○
Wellbutrin (Bupropion)	○
Other antidepressants - Adapin (Doxepin), Asendin (Amoxapine), Aventyl (Nortriptyline), Elavil (Amitriptyline, Endep), Ludiomil (Maprotiline), Marplan (Isocarboxazid), Pamelor (Nortriptyline), Pertrofrane (Protriptyline), Sinequan (Doxepin), Surmontil (Trimipramine), Trofranil (Imipramine), Vivactil (Protriptyline), Vestra (Reboxetine), Zispin (Mirtazapine):	○

Current Use: Anafranil Dose ______________________

Current Use: Date (Month/Year) Anafranil First Taken ______________________

Current Use: Date (Month/Day/Year) Anafranil Last Taken ______________________

Duration (Anafranil) - Months ______________________

Current Use: Desyrel Dose ______________________

Current Use: Date (Month/Year) Desyrel First Taken ______________________

Current Use: Date (Month/Day/Year) Desyrel Last Taken ______________________

Duration (Desyrel) - Months ______________________

Current Use: Nardil Dose ______________________

Current Use: Date (Month/Year) Nardil First Taken	______________
Current Use: Date (Month/Day/Year) Nardil Last Taken	______________
Duration (Nardil) - Months	______________
Current Use: Norpramine Dose	______________
Current Use: Date (Month/Year) Norpramine First Taken	______________
Current Use: Date (Month/Day/Year) Norpramine Last Taken	______________
Duration (Norpramine) - Months	______________
Current Use: Parnate Dose	______________
Current Use: Date (Month/Year) Parnate First Taken	______________
Current Use: Date (Month/Day/Year) Parnate Last Taken	______________
Duration (Parnate) - Months	______________
Current Use: Remeron Dose	______________
Current Use: Date (Month/Year) Remeron First Taken	______________
Current Use: Date (Month/Day/Year) Remeron Last Taken	______________
Duration (Remeron) - Months	______________
Current Use: Wellbutrin Dose	______________
Current Use: Date (Month/Year) Wellbutrin First Taken	______________
Current Use: Date (Month/Day/Year) Wellbutrin Last Taken	______________

Duration (Wellbutrin) - Months

____

Current Use: Other Dose

____

Current Use: Date (Month/Year) Other First Taken

____

Current Use: Date (Month/Day/Year) Other Last Taken

____

Duration (Other) - Months

____

Are you currently taking Lithium?
If Yes, ask the participant to specify which kind (Eskalith, Lithobid, or other).

	Current Use
Lithium (Eskalith)	○
Lithium (Lithobid)	○
Lithium (other generic)	○

Current Use: Eskalith Dose

____

Current Use: Date (Month/Year) Eskalith First Taken

____

Current Use: Date (Month/Day/Year) Eskalith Last Taken

____

Duration (Eskalith) - Months

____

Current Use: Lithobid Dose

____

Current Use: Date (Month/Year) Lithobid First Taken

____

Current Use: Date (Month/Day/Year) Lithobid Last Taken

____

Duration (Lithobid) - Months

____

Current Use: Other Generic Dose

____

Current Use: Date (Month/Year) Other Generic First Taken

____

Current Use: Date (Month/Day/Year) Other Generic Last Taken ____________________

Duration (Other Generic) - Months ____________________

Are you taking any benzodiazepines, such as Valium, Xanax, Ativan, or Klonopin?

	Current Use
Ativan (Lorazepam)	○
Klonopin (Clonazepam)	○
Serax (Oxazepam)	○
Tranxene (Clorazepate)	○
Valium (Diazepam)	○
Xanax (Alprazolam)	○

Current Use: Ativan Dose ____________________

Current Use: Date (Month/Year) Ativan First Taken ____________________

Current Use: Date (Month/Day/Year) Ativan Last Taken ____________________

Duration (Ativan) - Months ____________________

Current Use: Klonopin Dose ____________________

Current Use: Date (Month/Year) Klonopin First Taken ____________________

Current Use: Date (Month/Day/Year) Klonopin Last Taken ____________________

Duration (Klonopin) - Months ____________________

Current Use: Serax Dose ____________________

Current Use: Date (Month/Year) Serax First Taken ____________________

Current Use: Date (Month/Day/Year) Serax Last Taken ____________________

Duration (Serax) - Months ____________________

Current Use: Tranxene Dose
____

Current Use: Date (Month/Year) Tranxene First Taken
____

Current Use: Date (Month/Day/Year) Tranxene Last Taken
____

Duration (Tranxene) - Months
____

Current Use: Valium Dose
____

Current Use: Date (Month/Year) Valium First Taken
____

Current Use: Date (Month/Day/Year) Valium Last Taken
____

Duration (Valium) - Months
____

Current Use: Xanax Dose
____

Current Use: Date (Month/Year) Xanax First Taken
____

Current Use: Date (Month/Day/Year) Xanax Last Taken
____

Duration (Xanax) - Months
____

Are you taking any anticonvulsant medications?
Anticonvulsants include Depakote, Lamictal, Gabapentin, Topomax and others.

	Current Use
Depakote (Divalproex Sodium)	○
Lamictal (Lamotrigine)	○
Neurontin (Gabapentin)	○
Tegretol (Carbamazepine)	○
Topomax (Topiramate)	○

Current Use: Depakote Dose
____

Current Use: Date (Month/Year) Depakote First Taken
____

Current Use: Date (Month/Day/Year) Depakote Last Taken ____________________

Duration (Depakote) - Months ____________________

Current Use: Lamictal Dose ____________________

Current Use: Date (Month/Year) Lamictal First Taken ____________________

Current Use: Date (Month/Day/Year) Lamictal Last Taken ____________________

Duration (Lamictal) - Months ____________________

Current Use: Neurontin Dose ____________________

Current Use: Date (Month/Year) Neurontin First Taken ____________________

Current Use: Date (Month/Day/Year) Neurontin Last Taken ____________________

Duration (Neurontin) - Months ____________________

Current Use: Tegretol Dose ____________________

Current Use: Date (Month/Year) Tegretol First Taken ____________________

Current Use: Date (Month/Day/Year) Tegretol Last Taken ____________________

Duration (Tegretol) - Months ____________________

Current Use: Topomax Dose ____________________

Current Use: Date (Month/Year) Topomax First Taken ____________________

Current Use: Date (Month/Day/Year) Topomax Last Taken ____________________

Duration (Topomax) - Months ____________________

Are you taking any antipsychotic medications?
Examples include Haldol, Navane, or Thorazine.

	Current Use
Haldol (Haloperidol)	◯
Navane (Thiothixene)	◯
Prolixin (Fluphenazine)	◯
Stelazine (Trifluoperazine)	◯
Thorazine (Chlorpromazine)	◯
Other Antipsychotics - Loxitane (Loxipine), Mellaril (Thioridazine), Orap (Pimozaide), Permitil (Fluphenazine), Serentil (Mesoridazine), Taractan (Chloprothixene), Trilafon (Perphenazine), Vesprin (Promazine), Moban (Molindone):	◯

Current Use: Haldol Dose ______________________

Current Use: Date (Month/Year) Haldol First Taken ______________________

Current Use: Date (Month/Day/Year) Haldol Last Taken ______________________

Duration (Haldol) - Months ______________________

Current Use: Navane Dose ______________________

Current Use: Date (Month/Year) Navane First Taken ______________________

Current Use: Date (Month/Day/Year) Navane Last Taken ______________________

Duration (Navane) - Months ______________________

Current Use: Prolixin Dose ______________________

Current Use: Date (Month/Year) Prolixin First Taken ______________________

Current Use: Date (Month/Day/Year) Prolixin Last Taken ______________________

Duration (Prolixin) - Months ____________________

Current Use: Stelazine Dose ____________________

Current Use: Date (Month/Year) Stelazine First Taken ____________________

Current Use: Date (Month/Day/Year) Stelazine Last Taken ____________________

Duration (Stelazine) - Months ____________________

Current Use: Thorazine Dose ____________________

Current Use: Date (Month/Year) Thorazine First Taken ____________________

Current Use: Date (Month/Day/Year) Thorazine Last Taken ____________________

Duration (Thorazine) - Months ____________________

Current Use: Other Dose ____________________

Current Use: Date (Month/Year) Other First Taken ____________________

Current Use: Date (Month/Day/Year) Other Last Taken ____________________

Duration (Other) - Months ____________________

How about any of the following antipsychotics: Abilify, Clorazil, Risperdal, Seroquel?

	Current Use
Abilify (Aripiprazole)	○
Clozaril (Clozapine)	○
Geodon (Ziprasidone)	○
Risperdal (Risperidone)	○
Seroquel (Quetiapine)	○
Zyprexa (Olanzapine)	○

Current Use: Abilify Dose ____________________

Current Use: Date (Month/Year) Abilify First Taken	______________________
Current Use: Date (Month/Day/Year) Abilify Last Taken	______________________
Duration (Abilify) - Months	______________________
Current Use: Clozaril Dose	______________________
Current Use: Date (Month/Year) Clozaril First Taken	______________________
Current Use: Date (Month/Day/Year) Clozaril Last Taken	______________________
Duration (Clozaril) - Months	______________________
Current Use: Geodon Dose	______________________
Current Use: Date (Month/Year) Geodon First Taken	______________________
Current Use: Date (Month/Day/Year) Geodon Last Taken	______________________
Duration (Geodon) - Months	______________________
Current Use: Risperdal Dose	______________________
Current Use: Date (Month/Year) Risperdal First Taken	______________________
Current Use: Date (Month/Day/Year) Risperdal Last Taken	______________________
Duration (Risperdal) - Months	______________________
Current Use: Seroquel Dose	______________________
Current Use: Date (Month/Year) Seroquel First Taken	______________________
Current Use: Date (Month/Day/Year) Seroquel Last Taken	______________________

Duration (Seroquel) - Months

____

Current Use: Zyprexa Dose

____

Current Use: Date (Month/Year) Zyprexa First Taken

____

Current Use: Date (Month/Day/Year) Zyprexa Last Taken

____

Duration (Zyprexa) - Months

____

Are you currently taking any stimulant medications? Examples include Adderall, Concerta, or RItalin.

	Current Use
Adderall (Dextroamphetamine/Amphetamine Mixture)	○
Adderall XR (Dextroamphetamine/ Amphetamine Mixture SR)	○
Concerta (Methylphenidate SR)	○
Cylert (Pemoline)	○
Dexedrine (Dextroamphetamine)	○
Focalin (Dexmethylphenidate)	○
Metadate ER (Methylphenidate SR)	○
Ritalin (Methylphenidate SR)	○
Strattera (Atomoxetine)	○

Current Use: Adderall Dose

____

Current Use: Date (Month/Year) Adderall First Taken

____

Current Use: Date (Month/Day/Year) Adderall Last Taken

____

Duration (Adderall) - Months

____

Current Use: Adderall XR Dose

____

Current Use: Date (Month/Year) Adderall XR First Taken

____

Current Use: Date (Month/Day/Year) Adderall XR Last Taken	______________
Duration (Adderall XR) - Months	______________
Current Use: Concerta Dose	______________
Current Use: Date (Month/Year) Concerta First Taken	______________
Current Use: Date (Month/Day/Year) Concerta Last Taken	______________
Duration (Concerta) - Months	______________
Current Use: Cylert Dose	______________
Current Use: Date (Month/Day/Year) Cylert Last Taken	______________
Current Use: Date (Month/Year) Cylert First Taken	______________
Duration (Cylert) - Months	______________
Current Use: Dexedrine Dose	______________
Current Use: Date (Month/Year) Dexedrine First Taken	______________
Current Use: Date (Month/Day/Year) Dexedrine Last Taken	______________
Duration (Dexedrine) - Months	______________
Current Use: Focalin Dose	______________
Current Use: Date (Month/Year) Focalin First Taken	______________
Current Use: Date (Month/Day/Year) Focalin Last Taken	______________
Duration (Focalin) - Months	______________

Current Use: Metadate ER Dose
____

Current Use: Date (Month/Year) Metadate ER First Taken
____

Current Use: Date (Month/Day/Year) Metadate ER Last Taken
____

Duration (Metadate ER) - Months
____

Current Use: Ritalin Dose
____

Current Use: Date (Month/Year) Ritalin First Taken
____

Current Use: Date (Month/Day/Year) Ritalin Last Taken
____

Duration (Ritalin) - Months
____

Current Use: Strattera Dose
____

Current Use: Date (Month/Year) Strattera First Taken
____

Current Use: Date (Month/Day/Year) Strattera Last Taken
____

Duration (Strattera) - Months
____

Do you currently take any sleep medications?
Common prescribed sleep medications include Ambian, Lunesta, Sonata, and others.

	Current Use
Ambien (Zolpidem)	○
Lunesta (Eszopiclone)	○
Rozerem (Ramelteon)	○
Sonata (Zaleplon)	○
Silenor (Doxepin)	○
Other	○

Please specify:
____

Current Use: Ambien Dose
____

Current Use: Date (Month/Year) Ambien First Taken
____

Current Use: Date (Month/Day/Year) Ambien Last Taken
____

Duration (Ambien) - Months
____

Current Use: Lunesta Dose
____

Current Use: Date (Month/Year) Lunesta First Taken
____

Current Use: Date (Month/Day/Year) Lunesta Last Taken
____

Duration (Lunesta) - Months
____

Current Use: Rozerem Dose
____

Current Use: Date (Month/Year) Rozerem First Taken
____

Current Use: Date (Month/Day/Year) Rozerem Last Taken
____

Duration (Rozerem) - Months
____

Current Use: Sonata Dose
____

Current Use: Date (Month/Year) Sonata First Taken
____

Current Use: Date (Month/Day/Year) Sonata Last Taken
____

Duration (Sonata) - Months
____

Current Use: Silenor Dose
____

Current Use: Date (Month/Year) Silenor First Taken
____

Current Use: Date (Month/Day/Year) Silenor Last Taken
____

Duration (Silenor) - Months ________________

Current Use: Other Dose ________________

Current Use: Date (Month/Year) Other First Taken ________________

Current Use: Date (Month/Day/Year) Other Last Taken ________________

Duration (Other) - Months ________________

Are you taking any Thyroid medication or Birth Control?

	Current Use
Thyroid Medication	○
Birth Control	○

Thyroid - Please specify type: ________________

Current Use: Thyroid Medication Dose ________________

Current Use: Date (Month/Year) Thyroid Medication First Taken ________________

Current Use: Date (Month/Day/Year) Thyroid Medication Last Taken ________________

Duration (Thyroid Medication) - Months ________________

Birth Control - Please specify type: ________________

Current Use: Date (Month/Year) Birth Control First Taken ________________

Current Use: Date (Month/Day/Year) Birth Control Last Taken ________________

Duration (Birth Control) - Months ________________

Are you currently taking any other medications that we didn't cover so far?

If you are currently taking medications and have them in front of you, we can review them now to make sure that we have captured everything.

If there are more than 3 Other medications, you can list in the Notes box below.

	Current Use
Other	○
Other	○
Other	○

Please specify: ______________________

Current Use: Other Dose ______________________

Current Use: Date (Month/Year) Other First Taken ______________________

Current Use: Date (Month/Day/Year) Other Last Taken ______________________

Duration (Other) - Months ______________________

Please specify: ______________________

Current Use: Other Dose ______________________

Current Use: Date (Month/Year) Other First Taken ______________________

Current Use: Date (Month/Day/Year) Other Last Taken ______________________

Duration (Other) - Months ______________________

Please specify: ______________________

Current Use: Other Dose ______________________

Current Use: Date (Month/Year) Other First Taken ______________________

Current Use: Date (Month/Day/Year) Other Last Taken ______________________

Duration (Other) - Months

____

Thank you for answering my questions and sharing information about current medication use. I'm going to complete this form now, based on the information you provided.

Summary Scores

Duration - Unmedicated

______________________ (Please enter duration in months.)

This needs to be manually calculated and entered by the rater.

Duration unmedication is intended to capture the amount of time that they don't have any medications in their system, so even if they are prescribed medications, but aren't taking them, take that into consideration when estimating duration medicated.

If the outcome is that they have never taken prescribed medication, please enter "N/A (Lifetime)"

Current Use - SSRIs

____

Current Use - SNRIs

____

Current Use - Other Antidepressants

____

Current Use - Lithium

____

Current Use - Benzodiazepines

____

Current Use - Anticonvulsants

____

Current Use - Typical Antipsychotics

____

Current Use - Atypical Antipsychotics

____

Current Use - Stimulants

____

Current Use - Sleep Medications

____

Current Use - Other

____

Current Use for Any Drug	________________
Medication Naive	________________
Medication History Notes<br><br>List any additional medications that don't have a place above here (e.g., if there are more than 3 Other medications to record). Include the same information here as you would document above, listing name of medication, dosage, date first taken, date last taken, and duration (in months).<br><br>Include all prescribed medications, even if not taken by mouth, as there are some psychiatric medications (e.g., injectable, patch) that we would like to capture.	____________________<br>(If none, enter N/A)
Today's date:<br>Please record end time	________________

Demographics

Study ID	__________________________
Survey 2% Complete	
Today's date:	__________________________
Date of Birth	__________________________
Age	__________________________
Assigned sex at birth: What sex were you assigned at birth, on your original birth certificate?	◯ Male<br>◯ Female
Current gender identity: How do you describe yourself?	◯ Male<br>◯ Female<br>◯ Transgender<br>◯ Do not identify as male, female or transgender
Transgender status: Some people describe themselves as transgender when they experience a different gender identity from their sex at birth. For example, a person who was born into a male body but who feels female or lives as a woman.<br><br>Do you consider yourself to be transgender?	◯ No<br>◯ Yes, transgender, male to female<br>◯ Yes, transgender, female to male<br>◯ Yes, transgender, gender nonconforming
Do you consider yourself to be:	◯ Asexual<br>◯ Bisexual<br>◯ Heterosexual or straight<br>◯ Homosexual, gay, or lesbian<br>◯ Queer<br>◯ Questioning or unsure<br>◯ An identity not listed<br>◯ I prefer not to answer
Please specify:	__________________________
Current marital status:	◯ Single; never married<br>◯ Living with partner<br>◯ Domestic partnership<br>◯ Married<br>◯ Separated<br>◯ Divorced<br>◯ Widowed<br>◯ Don't know<br>◯ Prefer not to answer

Current living situation	◯ Alone<br>◯ With partner for at least one year<br>◯ With partner and children<br>◯ In parent's or children's home<br>◯ Siblings or other family member's home<br>◯ With friend<br>◯ Asylum/homeless<br>◯ Other<br>◯ Prefer not to answer
Do you have children (under the age of 18) in your home?	◯ No<br>◯ Yes, 1<br>◯ Yes, 2<br>◯ Yes, 3<br>◯ Yes, 4 or more
Are any of these children under the age of 5?	◯ No<br>◯ Yes, 1<br>◯ Yes, 2<br>◯ Yes, 3<br>◯ Yes, 4 or more
Height (in inches)<br><br>For example, if you are 5 feet and 5 inches, enter 65 (which is (5 x 12 = 60) + 5)	________________________
Weight (in lbs)	________________________
BMI	________________________
What race do you consider yourself to be?	☐ White<br>☐ Black/African American<br>☐ Native American<br>☐ Alaska Native<br>☐ Native Hawaiian<br>☐ Guamanian<br>☐ Samoan<br>☐ Other Pacific Islander<br>☐ Asian Indian<br>☐ Chinese<br>☐ Filipino<br>☐ Japanese<br>☐ Korean<br>☐ Vietnamese<br>☐ Other Asian<br>☐ Some other race<br>☐ Don't know<br>☐ Prefer not to answer
Please specify.	________________________
Do you consider yourself Hispanic/Latino?	◯ No<br>◯ Yes<br>◯ Don't know<br>◯ Prefer not to answer

Current Zip Code:

____

Current Zip Code:

____

What is the highest level of education you have completed?

- ◯ Never attended
- ◯ 1st grade
- ◯ 2nd grade
- ◯ 3rd grade
- ◯ 4th grade
- ◯ 5th grade
- ◯ 6th grade
- ◯ 7th grade
- ◯ 8th grade
- ◯ 9th grade
- ◯ 10th grade
- ◯ 11th grade
- ◯ 12th grade, no diploma
- ◯ High school Graduate
- ◯ GED or equivalent
- ◯ Some college
- ◯ Associate's Degree; Occupational, Technical, or Vocational
- ◯ Associate's Degree; Academic program
- ◯ Bachelor's Degree (ex. BA, AB, BS, BBA)
- ◯ Master's Degree (ex. MA, MS, MEng, MEd, MBA)
- ◯ Professional Degree (MD, DDS, DVM, JD)
- ◯ Doctoral Degree (ex. PhD, EdD)
- ◯ Don't know
- ◯ Prefer not to answer

We would like to know about what you do -- are you working now, looking for work, retired, keeping house, a student, or what?

- ◯ Working now full time
- ◯ Working now part time
- ◯ Only temporarily laid off, sick leave or maternity leave
- ◯ Looking for work, unemployed
- ◯ Unemployed, not looking for work
- ◯ Retired
- ◯ Disabled, permanently or temporarily
- ◯ Keeping house
- ◯ Student
- ◯ Other

Please specify.

____

What kind of work do you currently do?

- ◯ General laborer (e.g., laborer, porter, janitor, bricklayer, farm hand, deck hand)
- ◯ Homemaker
- ◯ Care Giver or Nanny
- ◯ Unemployed
- ◯ Transportation trades (e.g., bus driver, taxi driver, truck driver, heavy equipment operator)
- ◯ Retired
- ◯ Customer service (e.g., cashier, retail sales, bank teller, flight attendant)
- ◯ Healthcare (e.g., pharmacist, nurse, therapist, dentist, doctor)
- ◯ Food service (e.g., chef, food prep, waiter/waitress, bus boy, grocer, butcher)
- ◯ Skilled trade (e.g., mechanic, carpenter, electrician, technician, plumber)
- ◯ Public safety (e.g., police officer, EMT personnel, firefighter, corrections officer)
- ◯ Administrative/Clerical (e.g., office manager, administrative assistant, office secretary)
- ◯ Self-employed/Business owner (e.g., restaurant owner, small business owner)
- ◯ Educator/Instructor
- ◯ Arts/Entertainment/Athletics
- ◯ First-line supervisor (e.g., foreman, shift supervisor, maintenance supervisor)
- ◯ Manager/Director/Executive
- ◯ Professional (e.g., accountant, scientist, engineer, consultant, information technology)
- ◯ Armed Forces
- ◯ Student

The next question is about your total family income in the last calendar year BEFORE TAXES. Income is important in analyzing the health information we collect. For example, with this information, we can learn whether persons in one income group use certain types of medical services more or less often than those in another group. Please be assured that, like all other information you have provided, these answers will be kept strictly confidential.

When answering these questions, please remember that by "combined family income," we mean your income PLUS the income of all family members living in this household (including cohabitating partners, and armed forces members living at home).

What is your best estimate of your combined family income from all sources, before taxes, in the last calendar year?

- ◯ Less than $10,000
- ◯ $10,000 - $19,999
- ◯ $20,000 - $39,999
- ◯ $40,000 - $59,999
- ◯ $60,000 - $99,999
- ◯ $100,000 +
- ◯ Don't know
- ◯ Prefer not to answer

What category best represents your total personal income, before taxes, from all sources?	◯ Less than $10,000<br>◯ $10,000 - $19,999<br>◯ $20,000 - $39,999<br>◯ $40,000 - $59,999<br>◯ $60,000 - $99,999<br>◯ $100,000 +<br>◯ Don't know<br>◯ Prefer not to answer
Are you a registered student at UCLA?	◯ Yes<br>◯ No
What is your current official enrollment status?	◯ Full-time<br>◯ Part-time
What is your current student status?	◯ Freshman<br>◯ Sophomore<br>◯ Junior<br>◯ Senior<br>◯ Graduate Student<br>◯ Professional Student (M.D., J.D., etc.)<br>◯ Other
Please specify:	__________________________________
Today's date:	__________________________________

QUICK INVENTORY OF DEPRESSIVE SYMPTOMATOLOGY (SELF-REPORT) (QIDS-SR 16)

Please circle the one response to each item that best describes you for the past seven days.

1. Falling asleep:

0 I never take longer than 30 minutes to fall asleep.

1 I take at least 30 minutes to fall asleep, less than half the time.

2 I take at least 30 minutes to fall asleep, more than half the time.

3 I take more than 60 minutes to fall asleep, more than half the time.

2. Sleep during the night:

0 I do not wake up at night.

1 I have a restless, light sleep with a few brief awakenings each night.

2 I wake up at least once a night, but I go back to sleep easily.

3 I awaken more than once a night and stay awake for 20 minutes or more, more than half the time.

3. Waking up too early:

0 Most of the time, I awaken no more than 30 minutes before I need to get up.

1 More than half the time, I awaken more than 30 minutes before I need to get up.

2 I almost always awaken at least one hour or so before I need to, but I go back to sleep eventually.

3 I awaken at least one hour before I need to, and can’t go back to sleep.

4. Sleeping too much:

0 I sleep no longer than 7–8 hours/night, without napping during the day.

1 I sleep no longer than 10 hours in a 24-hour period including naps.

2 I sleep no longer than 12 hours in a 24-hour period including naps.

3 I sleep longer than 12 hours in a 24-hour period including naps.

QUICK INVENTORY OF DEPRESSIVE SYMPTOMATOLOGY (SELF-REPORT) (QIDS-SR 16) (Continued)

Please circle the one response to each item that best describes you for the past seven days.

5. Feeling sad:

0 I do not feel sad.

1 I feel sad less than half the time.

2 I feel sad more than half the time.

3 I feel sad nearly all of the time.

6. Decreased appetite:

0 There is no change in my usual appetite.

1 I eat somewhat less often or lesser amounts of food than usual.

2 I eat much less than usual and only with personal effort.

3 I rarely eat within a 24-hour period, and only with extreme personal effort or when others persuade me to eat.

7. Increased appetite:

0 There is no change from my usual appetite.

1 I feel a need to eat more frequently than usual.

2 I regularly eat more often and/or greater amounts of food than usual.

3 I feel driven to overeat both at mealtime and between meals.

8. Decreased weight (within the last two weeks):

0 I have not had a change in my weight.

1 I feel as if I've had a slight weight loss.

2 I have lost 2 pounds or more.

3 I have lost 5 pounds or more.

9. Increased weight (within the last two weeks):

0 I have not had a change in my weight.

1 I feel as if I've had a slight weight gain.

2 I have gained 2 pounds or more.

3 I have gained 5 pounds or more.

QUICK INVENTORY OF DEPRESSIVE SYMPTOMATOLOGY (SELF-REPORT) (QIDS-SR 16) (Continued)

Please circle the one response to each item that best describes you for the past seven days.

10. Concentration/Decision making:

0	There is no change in my usual capacity to concentrate or make decisions.
1	I occasionally feel indecisive or find that my attention wanders.
2	Most of the time, I struggle to focus my attention or to make decisions.
3	I cannot concentrate well enough to read or cannot make even minor decisions.

11. View of myself:

0	I see myself as equally worthwhile and deserving as other people.
1	I am more self-blaming than usual.
2	I largely believe that I cause problems for others.
3	I think almost constantly about major and minor defects in myself.

12. General interest:

0	There is no change from usual in how interested I am in other people or activities.
1	I notice that I am less interested in people or activities.
2	I find I have interest in only one or two of my formerly pursued activities.
3	I have virtually no interest in formerly pursued activities.

13. Energy level:

0	There is no change in my usual level of energy.
1	I get tired more easily than usual.
2	I have to make a big effort to start or finish my usual daily activities (for example, shopping, homework, cooking or going to work).
3	I really cannot carry out most of my usual daily activities because I just don’t have the energy.

QUICK INVENTORY OF DEPRESSIVE SYMPTOMATOLOGY (SELF-REPORT) (QIDS-SR 16) (Continued)

Please circle the one response to each item that best describes you for the past seven days.

14. Feeling slowed down:

0	I think, speak, and move at my usual rate of speed.
1	I find that my thinking is slowed down or my voice sounds dull or flat.
2	It takes me several seconds to respond to most questions and I'm sure my thinking is slowed.
3	I am often unable to respond to questions without extreme effort.

15. Feeling restless:

0	I do not feel restless.
1	I'm often fidgety, wringing my hands, or need to shift how I am sitting.
2	I have impulses to move about and am quite restless.
3	At times, I am unable to stay seated and need to pace around.

How Irritable are You? (Brief Irritability Test, BITe)

Introduction

Irritability is different from trait anger. It is the proneness and susceptibility towards states of frustration and annoyance despite little provocation. This is sometimes expressed outwardly (Holtzman et al. 2015). This very short 5-item test assesses your irritability. Quite fittingly, this is a sufficiently short measure if you are irritated by long questionnaires!

	Never	Rarely	Sometimes	Often	Very Often	Always
**1. I have been grumpy.**						
**2. I have been feeling like I might snap.**						
**3. Other people have been getting on my nerves.**						
**4. Things have been bothering me more than they normally do.**						
**5. I have been feeling irritable.**						

Scoring

Never = 1, Rarely = 2, Sometimes = 3, Often = 4, Very Often = 5, Always = 6

Total irritability score = Sum items 1-5

Interpretation

A sample of 1,116 men and women report an average score of 12.69 for men, and 13.13 for women in terms of irritability.

Reference

Holtzman, S., O'Connor, B. P., Barata, P. C., & Stewart, D. E. (2015). The Brief Irritability Test (BITe) A Measure of Irritability for Use Among Men and Women. *Assessment*, *22*(1), 101-115.

Lifetime and Current Suicidal Ideation Assessment - Self Report

**1.** Have you <u>ever</u> purposely hurt yourself without wanting to die? We are referring to examples like cutting or burning your skin to hurt yourself but without wanting to die.<br><br>We are <u>not</u> asking about times where you have <u>indirectly</u> hurt yourself, for example times where you starved yourself, overdosed without wanting to die, got a tattoo/piercing.	☐ no (skip to 3)<br><br>☐ yes
**2.** How many episodes in the <u>past two weeks</u> have you purposely hurt yourself without wanting to die? (give your best estimate)	# of episodes<br><br>___________
**3.** Have you <u>ever</u> said or done something to purposely lead someone to believe that you wanted to kill yourself when you really had no intention of dying?	☐ no (skip to 5)<br><br>☐ yes
**4.** In the <u>past two weeks</u> have you purposely led someone to believe that you wanted to kill yourself when you really had no intention of dying?	☐ no<br>☐ yes
**5.** Have you <u>ever</u> actually had any thoughts about killing yourself?	☐ no<br>☐ yes
**6.** Please select each of the following thoughts you've ever had<br>☐ "I wish I could disappear or not exist"<br>☐ "I wish I were never born"<br>☐ "My life is not worth living"<br>☐ "I wish I would go to sleep and never wake up again"<br>☐ "I wish I were dead"<br>☐ "Maybe I should kill myself"<br>☐ "I should kill myself"<br>☐ "I am going to kill myself"<br>☐ None of these (skip to 8)	
**7.** In the <u>past two weeks</u>, did you actually have thoughts about killing yourself?	☐ no<br><br>☐ yes

Lifetime and Current Suicidal Ideation Assessment - Self Report

**8.**	Have you ever thought about how you might kill yourself?<br><br>*(ex. "I thought about taking an overdose **but** I never worked out the details about when, where, and how I would do that and I would never act on these thoughts.")*	☐ no (skip to 12)<br><br>☐ yes
**9.**	In the past two weeks, did you have thoughts about how you might kill yourself?<br><br>*(ex. "I thought about taking an overdose **but** I never worked out the details about when, where, and how I would do that and I would never act on these thoughts.")*	☐ no<br><br>☐ yes
**10.**	Have you ever had any intention of acting on thoughts of killing yourself, as opposed to you have the thoughts but you definitely would not act on them?<br>*(ex. "I thought about killing myself by taking an overdose **and** considered going through with it.")*	☐ no<br><br>☐ yes
**11.**	In the past two weeks, have you had any intention of acting on thoughts of killing yourself, as opposed to you have the thoughts but you definitely would not act on them?<br>*(ex. "I had the thought of killing myself by taking an overdose **and** considered going through with it.")*	☐ no<br><br>☐ yes
**12.**	Have you ever started to work out, or actually worked out, the specific details of how to kill yourself **and** did you ever actually intend to carry out the details of your plan?<br><br>*(ex. "I was planning to take 3 bottles of my sleep medication on a Saturday when no one was around to stop me.")*	☐ no (skip to 14)<br><br>☐ yes
**13.**	In the past two weeks have you started to work out, or actually worked out, the specific details of how to kill yourself **and** did you actually intend to carry out the details of your plan?<br><br>*(ex. "I am planning to take 3 bottles of my sleep medication this Saturday when no one is around to stop me.")*	☐ no<br><br>☐ yes
**14.**	Have you ever done anything, started to do anything, or prepared to do anything to end your life?<br><br>*(ex. collected pills, obtained a gun, gave away valuables, wrote a will or suicide note, etc.)*	☐ no (skip to 16)<br><br>☐ yes

Lifetime and Current Suicidal Ideation Assessment - Self Report

**15.** In the past two weeks have you done anything, started to do anything, or prepared to do anything to end your life?<br><br>*(ex. collected pills, obtained a gun, gave away valuables, wrote a will or suicide note, etc.)*	☐ no<br><br>☐ yes
**16.** Have you ever been very close to killing yourself and at the last minute, something or someone else stopped you, or you decided not to *before you took any action*?<br><br>*(ex. held a bottle of pills in your hand but decided not to take any, set up a noose but decided not to use it, pointed a gun to your head, but decided not to use it)?*	☐ no (skip to 19)<br><br>☐ yes
**17.** How many times in your lifetime have you been very close to killing yourself and at the last minute, something or someone else stopped you, or you decided not to *before you took any action*? (give your best estimate)	#of times<br><br>__________
**18.** How many times in the past two weeks have you been very close to killing yourself and at the last minute, something or someone else stopped you, or you decided not to *before you took any action*? (give your best estimate)	#of times<br><br>___________
**19.** Have you ever tried to kill yourself?	☐ no (skip to 22)<br><br>☐ yes
**20.** How many times have you tried to kill yourself in your lifetime**?**	#of attempts<br><br>__________
**21.** How many times have you tried to kill yourself in the past year**?**	#of attempts<br><br>__________

22. On a scale of 0 to 4, what do you think the likelihood is that you will try to kill yourself in the future?

☐ **0**	☐ **1**	☐ **2**	☐ **3**	☐ **4**
**Not at all**	**A little bit**	**Somewhat**	**Very Much**	**Extremely**

Generalized Anxiety Disorder Screener (GAD-7)

Over the *last 2 weeks*, how often have you been bothered by the following problems?	Not at all	Several Days	More than half the days	Nearly every day
1. Feeling nervous, anxious or on edge	0	1	2	3
2. Not being able to stop or control worrying	0	1	2	3
3. Worrying too much about different things	0	1	2	3
4. Trouble relaxing	0	1	2	3
5. Being so restless that it is hard to sit still	0	1	2	3
6. Becoming easily annoyed or irritated	0	1	2	3
7. Feeling afraid as if something awful might happen	0	1	2	3
	Add columns			
	Total Score			
8. If you checked off any problems, how difficult have these problems made it for you to do your work, take care of things at home, or get along with other people?	Not difficult at all	Somewhat difficult	Very difficult	Extremely difficult

When did the symptoms begin? ____

Generalized Anxiety Disorder Screener (GAD-7)

Scoring and Interpretation:

GAD-2 Score*	Provisional Diagnosis
0-2	None
3-6	Probable anxiety disorder
**GAD-7 Score**	**Provisional Diagnosis**
0-7	None
8+	Probable anxiety disorder

*GAD-2 is the first 2 questions of the GAD-7

References:

- Spitzer RL, Kroenke K, Williams JB, Lowe B. A brief measure for assessing generalized anxiety disorder: the GAD-7. Archives of internal medicine. May 22 2006;166(10):1092-1097. PMID: 16717171
- Kroenke K, Spitzer RL, Williams JB, Monahan PO, Lowe B. Anxiety disorders in primary care: prevalence, impairment, comorbidity, and detection. Annals of internal medicine. Mar 6 2007;146(5):317-325. PMID: 17339617
- Lowe B, Decker O, Muller S, et al. Validation and standardization of the Generalized Anxiety Disorder Screener (GAD-7) in the general population. Medical care. Mar 2008;46(3):266-274. PMID: 18388841

Work and Social Adjustment Scale (WSAS)

Mental health can affect one's ability to do certain day-to-day tasks in their lives. Please read each item below and respond based on how much your mental health impairs your ability to carry out the activity.

		Not at All		Slightly		Definitely		Markedly		Very Severely
1.	Because of my mental health my **ability to work** is impaired. '0' means 'not at all impaired' and '8' means very severely impaired to the point I can't work.	0	1	2	3	4	5	6	7	8
2.	Because of my mental health my **home management** (cleaning, tidying, shopping, cooking, looking after home or children, paying bills) is impaired.	0	1	2	3	4	5	6	7	8
3.	Because of my mental health my **social leisure activities** (with other people e.g. parties, bars, clubs, outings, visits, dating, home entertaining) are impaired.	0	1	2	3	4	5	6	7	8
4.	Because of my mental health, my **private leisure activities** (done alone, such as reading, gardening, collecting, sewing, walking alone) are impaired.	0	1	2	3	4	5	6	7	8
5.	Because of my mental health, my ability to form and maintain **close relationships** with others, including those I live with, is impaired.	0	1	2	3	4	5	6	7	8

Copyright Information:

Mundt, J. C., I. M. Marks, et al. (2002). "The Work and Social Adjustment Scale: A simple measure of impairment in functioning." Br. J. Psychiatry 180: 461-4. Reproduced with the kind permission of Professor Isaac Marks.

Psychiatric Research Unit
WHO Collaborating Centre in Mental Health

WHO (Five) Well-Being Index (1998 version)

Please indicate for each of the five statements which is closest to how you have been feeling over the last two weeks. Notice that higher numbers mean better well-being.

Example: If you have felt cheerful and in good spirits more than half of the time during the last two weeks, put a tick in the box with the number 3 in the upper right corner.

Over the last two weeks:	All the time	Most of the time	More than half of the time	Less than half of the time	Some of the time	At no time
**1. I have felt cheerful and in good spirits**	5	4	3	2	1	0
**2. I have felt calm and relaxed**	5	4	3	2	1	0
**3. I have felt active and vigorous**	5	4	3	2	1	0
**4. I woke up feeling fresh and rested**	5	4	3	2	1	0
**5. My daily life has been filled with things that interest me**	5	4	3	2	1	0

Scoring:

The raw score is calculated by totaling the figures of the five answers. The raw score ranges from 0 to 25, 0 represent- ing worst possible and 25 representing best possible quality of life.

To obtain a percentage score ranging from 0 to 100, the raw score is multiplied by 4. A percentage score of 0 represents worst possible, whereas a score of 100 represents best possible quality of life.

Interpretation:

It is recommended to administer the Major Depression (ICD-10) Inventory if the raw score is below 13 or if the patient has answered 0 to 1 to any of the five items. A score below 13 indicates poor wellbeing and is an indication for testing for depression under ICD-10.

Monitoring change:

In order to monitor possible changes in wellbeing, the percentage score is used. A 10% difference indicates a significant change (ref. John Ware, 1995).

© Psychiatric Research Unit, WHO Collaborating Center for Mental Health, Frederiksborg General Hospital, DK-3400 Hillerød

Subject's Initials________________ID#__________________Date_______________Time_____ AM/PM

PITTSBURGH SLEEP QUALITY INDEX

INSTRUCTIONS:
The following questions relate to your usual sleep habits during the past month only. Your answers should indicate the most accurate reply for the majority of days and nights in the past month. Please answer all questions.

---

1. During the past month, what time have you usually gone to bed at night?

   BED TIME ___________

2. During the past month, how long (in minutes) has it usually taken you to fall asleep each night?

   NUMBER OF MINUTES ___________

3. During the past month, what time have you usually gotten up in the morning?

   GETTING UP TIME ___________

4. During the past month, how many hours of actual sleep did you get at night? (This may be different than the number of hours you spent in bed.)

   HOURS OF SLEEP PER NIGHT ___________

For each of the remaining questions, check the one best response. Please answer all questions.

5. During the past month, how often have you had trouble sleeping because you . . .

a) Cannot get to sleep within 30 minutes

Not during the past month_____ Less than once a week_____ Once or twice a week_____ Three or more times a week_____

b) Wake up in the middle of the night or early morning

Not during the past month_____ Less than once a week_____ Once or twice a week_____ Three or more times a week_____

c) Have to get up to use the bathroom

Not during the past month_____ Less than once a week_____ Once or twice a week_____ Three or more times a week_____

d) Cannot breathe comfortably

Not during the past month_____	Less than once a week_____	Once or twice a week_____	Three or more times a week_____

e) Cough or snore loudly

Not during the past month_____	Less than once a week_____	Once or twice a week_____	Three or more times a week_____

f) Feel too cold

Not during the past month_____	Less than once a week_____	Once or twice a week_____	Three or more times a week_____

g) Feel too hot

Not during the past month_____	Less than once a week_____	Once or twice a week_____	Three or more times a week_____

h) Had bad dreams

Not during the past month_____	Less than once a week_____	Once or twice a week_____	Three or more times a week_____

i) Have pain

Not during the past month_____	Less than once a week_____	Once or twice a week_____	Three or more times a week_____

j) Other reason(s), please describe____

____

How often during the past month have you had trouble sleeping because of this?

Not during the past month_____	Less than once a week_____	Once or twice a week_____	Three or more times a week_____

6. During the past month, how would you rate your sleep quality overall?

Very good ___________

Fairly good ___________

Fairly bad ___________

Very bad ___________

7. During the past month, how often have you taken medicine to help you sleep (prescribed or "over the counter")?

Not during the past month_____	Less than once a week_____	Once or twice a week_____	Three or more times a week_____

8. During the past month, how often have you had trouble staying awake while driving, eating meals, or engaging in social activity?

Not during the past month_____	Less than once a week_____	Once or twice a week_____	Three or more times a week_____

9. During the past month, how much of a problem has it been for you to keep up enough enthusiasm to get things done?

No problem at all	__________
Only a very slight problem	__________
Somewhat of a problem	__________
A very big problem	__________

10. Do you have a bed partner or room mate?

No bed partner or room mate	__________
Partner/room mate in other room	__________
Partner in same room, but not same bed	__________
Partner in same bed	__________

If you have a room mate or bed partner, ask him/her how often in the past month you have had . . .

a) Loud snoring

Not during the past month_____	Less than once a week_____	Once or twice a week_____	Three or more times a week_____

b) Long pauses between breaths while asleep

Not during the past month_____	Less than once a week_____	Once or twice a week_____	Three or more times a week_____

c) Legs twitching or jerking while you sleep

Not during the past month_____	Less than once a week_____	Once or twice a week_____	Three or more times a week_____

d) Episodes of disorientation or confusion during sleep

Not during the past month_____	Less than once a week_____	Once or twice a week_____	Three or more times a week_____

e) Other restlessness while you sleep; please describe__________________________________

____

Not during the past month_____	Less than once a week_____	Once or twice a week_____	Three or more times a week_____

© 1989, University of Pittsburgh. All rights reserved. Developed by Buysse,D.J., Reynolds,C.F., Monk,T.H., Berman,S.R., and Kupfer,D.J. of the University of Pittsburgh using National Institute of Mental Health Funding.

Buysse DJ, Reynolds CF, Monk TH, Berman SR, Kupfer DJ: Psychiatry Research, 28:193-213, 1989.

Snaith-Hamilton Pleasure Scale

Study ID ______________________

This questionnaire is designed to measure your ability to experience pleasure in the last few days. It is important to read each statement very carefully. Indicate how much you agree or disagree with each statement.

	Strongly Disagree	Disagree	Agree	Strongly Agree
I would enjoy watching shows, or listing to the radio or podcasts	○	○	○	○
I would enjoy being with my family or close friends	○	○	○	○
I would find pleasure in my hobbies and pastimes	○	○	○	○
I would be able to enjoy my favorite meal	○	○	○	○
I would enjoy a warm bath or refreshing shower	○	○	○	○
I would find pleasure in the scent of flowers, the smell of a fresh sea breeze, or the smell of freshly baked bread	○	○	○	○
I would enjoy seeing other people's smiling faces	○	○	○	○
I would enjoy looking sharp when I have made an effort with my appearance	○	○	○	○
I would enjoy reading a book, magazine or newspaper	○	○	○	○
I would enjoy a cup of tea or coffee, or my favorite drink	○	○	○	○
I would find pleasure in small things, e.g., bright sunny day, a telephone call from a friend	○	○	○	○
I would be able to enjoy a beautiful landscape or view	○	○	○	○
I would get pleasure from helping others	○	○	○	○
I would feel pleasure when I receive praise from other people	○	○	○	○

Total Sum ______________________

Threshold Score ______________________

Below are a number of statements. Each statement asks you to think about your life over the last 2 weeks.
For each statement, select how appropriately it describes your life right now. Select "Completely true" if the statement describes you perfectly, "Completely untrue" if the statement does not describe you at all over the last 2 weeks, and use the answers in between accordingly.

		Completely UNTRUE	Mostly untrue	Neither true nor untrue	Quite true	Completely TRUE
1	I feel sad or upset when I hear bad news.	☐	☐	☐	☐	☐
2	I start conversations with random people.	☐	☐	☐	☐	☐
3	I enjoy doing things with people I have just met.	☐	☐	☐	☐	☐
4	I suggest activities for me and my friends to do.	☐	☐	☐	☐	☐
5	I make decisions firmly and without hesitation.	☐	☐	☐	☐	☐
6	After making a decision, I will wonder if I have made the wrong choice.	☐	☐	☐	☐	☐
7	Based on the last two weeks, I would say I care deeply about how my loved ones think of me.	☐	☐	☐	☐	☐
8	I go out with friends on a weekly basis.	☐	☐	☐	☐	☐
9	When I decide to do something, I am able to make an effort easily.	☐	☐	☐	☐	☐
10	I don't like to laze around.	☐	☐	☐	☐	☐
11	I get things done when they need to be done, without requiring reminders from others.	☐	☐	☐	☐	☐
12	When I decide to do something, I am motivated to see it through to the end.	☐	☐	☐	☐	☐
13	I feel awful if I say something insensitive.	☐	☐	☐	☐	☐
14	I start conversations without being prompted.	☐	☐	☐	☐	☐
15	When I have something I need to do, I do it straightaway so it is out of the way.	☐	☐	☐	☐	☐
16	I feel bad when I hear an acquaintance has an accident or illness.	☐	☐	☐	☐	☐
17	I enjoy choosing what to do from a range of activities.	☐	☐	☐	☐	☐
18	If I realise I have been unpleasant to someone, I will feel terribly guilty afterwards.	☐	☐	☐	☐	☐

Scoring Instructions

Each item is negatively scored i.e. you will need to REVERSE ALL ITEMS:
Completely TRUE = 0
Quite true = 1
Neither true nor untrue = 2
Mostly untrue = 3
Completely UNTRUE = 4

Three domains of apathy-motivation are assessed with the mean score, which ranges from 0-4 (with 0 being motivated and 4 being apathetic).

(1) Behavioural: Q5, 9, 10, 11, 12, 15
(2) Social: Q2, 3, 4, 8, 14, 17
(3) Emotional: Q1, 6, 7, 13, 16, 18

RRS Info for WL

Ruminative Response Style Questionnaire – Brooding Subscale – 9-item (RRS-Brooding)

Updated baseline (e.g. “trait”) RRS-Brooding:

People think and do many different things when they feel depressed. Please read each of the items below and indicate whether you almost never, sometimes, often, or almost always think or do each one when you feel down, sad, or depressed. Please indicate what you *generally* do, not what you think you should do.

	Almost never	Sometimes	Often	Almost Always
1) Think ‘What am I doing to deserve this?’	☐	☐	☐	☐
2) Think ‘Why do I always react this way?’	☐	☐	☐	☐
3) Think about a recent situation, wishing it had gone better	☐	☐	☐	☐
4) Think ‘Why do I have problems other people don’t have?’	☐	☐	☐	☐
5) Think ‘Why can’t I handle things better?’	☐	☐	☐	☐
6) Think about how alone you feel	☐	☐	☐	☐
7) Think about how sad you feel	☐	☐	☐	☐
8) Think about all your shortcomings, failings, faults, mistakes	☐	☐	☐	☐
9) I try to understand myself by focusing on my depressed feelings.	☐	☐	☐	☐

Updated weekly RRS-Brooding will look like this (to be used at Week 6 and Week 12):

People think and do many different things when they feel down, sad or depressed. Please read each of the items below and indicate if you almost never, sometimes, often, or almost always thought or did what is described in each statement over the last week. Please indicate what you did *over the last week*, not what you think you should have done.

	Almost never	Sometimes	Often	Almost Always
1) Think about how alone you feel	☐	☐	☐	☐
2) Think ‘Why do I have problems other people don’t have?’	☐	☐	☐	☐
3) Think about how sad you feel	☐	☐	☐	☐
4) Think about all your shortcomings, failings, faults, mistakes	☐	☐	☐	☐
5) I try to understand myself by focusing on my depressed feelings.	☐	☐	☐	☐
6) Think ‘What am I doing to deserve this?’	☐	☐	☐	☐
7) Think ‘Why do I always react this way?’	☐	☐	☐	☐
8) Think about a recent situation, wishing it had gone better	☐	☐	☐	☐
9) Think ‘Why can’t I handle things better?’	☐	☐	☐	☐

Eudaimonic Well-Being Questionnaire

Instructions: Using the 0-4 scale below, indicate the extent to which you have felt the following ways **over the past week**.

Not at all	A little bit	Moderately	Quite a bit	Extremely
0	1	2	3	4

1. How much do you feel your life has a sense of meaning?
2. How much do you feel a sense of purpose in life?
3. How much do you feel connected to your values?
4. How much do you feel like you have warm and trusting relationships with others?
5. How much do you feel confident and positive about yourself?

Adapted from ***Mental Health Continuum Short Form (MCH-SF; Keyes et al., 2008)*** and ***Daily Meaning Scale (DMS; Steger, Kashdan & Oishi, 2008)***

U.S. Household Food Security Survey Module: Six-Item Short Form
Economic Research Service, USDA
September 2012

Revision Notes: The food security questions in the 6-item module are essentially unchanged from those in the original module first implemented in 1995 and described previously in this document.

September 2012:

- Added coding specification for "How many days" for 30-day version of AD1a.

July 2008:

- Wording of resource constraint in AD2 was corrected to, "…because there wasn't enough money for food" to be consistent with the intention of the September 2006 revision.

January 2008:

- Corrected user notes for coding AD1a.

September 2006:

- Minor changes were introduced to standardize wording of the resource constraint in most questions to read, "…because there wasn't enough money for food."
- Question numbers were changed to be consistent with those in the revised Household Food Security Survey Module.
- User notes following the questionnaire were revised to be consistent with current practice and with new labels for ranges of food security and food insecurity introduced by USDA in 2006.

Overview: The six-item short form of the survey module and the associated Six-Item Food Security Scale were developed by researchers at the National Center for Health Statistics.

Background: The six-item short form of the survey module and the associated Six-Item Food Security Scale were developed by researchers at the National Center for Health Statistics in collaboration with Abt Associates Inc. and documented in "The effectiveness of a short form of the household food security scale," by S.J. Blumberg, K. Bialostosky, W.L. Hamilton, and R.R. Briefel (published by the *American Journal of Public Health*, vol. 89, pp. 1231-34, 1999). ERS conducted additional assessment of classification sensitivity, specificity, and bias relative to the 18-item scale.

If respondent burden permits, use of the 18-item U.S. Household Food Security Survey Module or the 10-item U.S. Adult Food Security Survey Module is recommended. However, in surveys that cannot implement one of those measures, the six-item module may provide an acceptable substitute. It has been shown to identify food-insecure households and households with very low food security with reasonably high specificity and sensitivity and minimal bias compared with the 18-item measure. It does not, however, directly ask about children's food security, and does not measure the most severe range of adult food insecurity, in which children's food intake is likely to be reduced.

[Begin Six-Item Food Security Module]

<u>Transition into Module</u>:
These next questions are about the food eaten in your household in the last 12 months, since (current month) of last year and whether you were able to afford the food you need.

<u>NOTE:</u> If the placement of these items in the survey makes the transition/introductory sentence unnecessary, add the word "Now" to the beginning of question HH3: "Now I'm going to read you...."

FILL INSTRUCTIONS: Select the appropriate fill from parenthetical choices depending on the number of persons and number of adults in the household.

HH3. I'm going to read you several statements that people have made about their food situation. For these statements, please tell me whether the statement was <u>often</u> true, <u>sometimes</u> true, or <u>never</u> true for (you/your household) in the last 12 months—that is, since last (name of current month).

The first statement is, "The food that (I/we) bought just didn't last, and (I/we) didn't have money to get more." Was that <u>often</u>, <u>sometimes</u>, or <u>never</u> true for (you/your household) in the last 12 months?

[ ] Often true
[ ] Sometimes true
[ ] Never true
[ ] DK or Refused

HH4. "(I/we) couldn't afford to eat balanced meals." Was that <u>often</u>, <u>sometimes</u>, or <u>never</u> true for (you/your household) in the last 12 months?

[ ] Often true
[ ] Sometimes true
[ ] Never true
[ ] DK or Refused

AD1. In the last 12 months, since last (name of current month), did (you/you or other adults in your household) ever cut the size of your meals or skip meals because there wasn't enough money for food?

[ ] Yes
[ ] No (Skip AD1a)
[ ] DK (Skip AD1a)

AD1a. [IF YES ABOVE, ASK] How often did this happen—almost every month, some months but not every month, or in only 1 or 2 months?

[ ] Almost every month
[ ] Some months but not every month
[ ] Only 1 or 2 months
[ ] DK

AD2. In the last 12 months, did you ever eat less than you felt you should because there wasn't enough money for food?

[ ] Yes
[ ] No
[ ] DK

AD3. In the last 12 months, were you every hungry but didn't eat because there wasn't enough money for food?

[ ] Yes
[ ] No
[ ] DK

[End of Six-Item Food Security Module]

User Notes

<u>(1) Coding Responses and Assessing Households' Food Security Status:</u>

Responses of "often" or "sometimes" on questions HH3 and HH4, and "yes" on AD1, AD2, and AD3 are coded as affirmative (yes). Responses of "almost every month" and "some months but not every month" on AD1a are coded as affirmative (yes). The sum of affirmative responses to the six questions in the module is the household's raw score on the scale.

Food security status is assigned as follows:

- Raw score 0-1—High or marginal food security (raw score 1 may be considered marginal food security, but a large proportion of households that would be measured as having marginal food security using the household or adult scale will have raw score zero on the six-item scale)
- Raw score 2-4—Low food security
- Raw score 5-6—Very low food security

For some reporting purposes, the food security status of households with raw score 0-1 is described as food secure and the two categories "low food security" and "very low food security" in combination are referred to as food insecure.

For statistical procedures that require an interval-level measure, the following scale scores, based on the Rasch measurement model may be used:

Number of affirmatives	Scale score
0	NA
1	2.86
2	4.19
3	5.27
4	6.30
5	7.54
6<br>(evaluated at 5.5)	8.48

However, no interval-level score is defined for households that affirm no items. (They are food secure, but the extent to which their food security differs from households that affirm one item is not known.)

<u>(2) Response Options:</u> For interviewer-administered surveys, DK ("don't know") and "Refused" are blind responses—that is, they are not presented as response options but marked if volunteered. For self-administered surveys, "don't know" is presented as a response option.

(3) Screening: If it is important to minimize respondent burden, respondents may be screened after question AD1. Households that have responded "never" to HH3 and HH4 and "no" to AD1 may skip over the remaining questions and be assigned raw score zero. In pilot surveys intended to validate the module in a new cultural, linguistic, or survey context, however, screening should be avoided if possible and all questions should be administered to all respondents.

(4) 30-Day Reference Period: The questionnaire items may be modified to a 30-day reference period by changing the "last 12-month" references to "last 30 days." In this case, item AD1a must be changed to read as follows:

AD1a. [IF YES ABOVE, ASK] In the last 30 days, how many days did this happen?

______ days

[ ] DK

Responses of 3 days or more are coded as "affirmative" responses.

(5) Self Administration: The six-item module has been used successfully in mail-out, take-home, and on-site self-administered surveys. For self-administration, question AD1a may be presented in one of two ways:

- Indent AD1a below AD1 and direct the respondent to AD1a with an arrow from the "Yes" response box of AD1. In a parenthetical following the "No" response box of AD1, instruct the respondent to skip question AD1 and go to question AD2.
- Present the following response options to question AD1 and omit question AD1a:
  - Yes, almost every month
  - Yes, some months but not every month
  - Yes, only 1 or 2 months
  - No

  In this case, either of the first two responses is scored as two affirmative responses, while "Yes, only 1 or 2 months" is scored as a single affirmative response.

The two approaches have been found to yield nearly equal results. The latter may be preferred because it usually reduces the proportion of respondents with missing information on how often this behavior occurred.

Comorbidity Questionnaire

National Network of Depression Centers (NNDC) self-administered Comorbidity Screener records 15 medically-relevant conditions (e.g., thyroid disease) to depression.

We created a Comorbidity Load score based on responses to items 1-15. All entries are either 0(No) or 1(Yes).
Sum total Yes responses.
Possible range of scores 0 - 15.

Today's date: ______________________

The following is a list of common problems. Please indicate if you currently have the problem. If you do, you will be asked to indicate if you receive medications or some other type of treatment for the problem and if the problem limits any of your activities.

Do you have the problem?

	No	Yes
Heart disease	◯	◯
High blood pressure	◯	◯
Lung disease	◯	◯
Diabetes	◯	◯
Ulcer or stomach disease	◯	◯
Kidney disease	◯	◯
Liver disease	◯	◯
Anemia or other blood disease	◯	◯
Cancer	◯	◯
Seizure disorder (epilepsy)	◯	◯
Other neurological disease	◯	◯
Thyroid disease	◯	◯
Osteoarthritis, degenerative arthritis	◯	◯
Back pain	◯	◯
Rheumatoid arthritis	◯	◯

Please indicate other neurological diseases. ______________________

Do you receive treatment for it?

	No	Yes
Heart disease	◯	◯
High blood pressure	◯	◯
Lung disease	◯	◯

Diabetes	◯	◯
Ulcer or stomach disease	◯	◯
Kidney disease	◯	◯
Liver disease	◯	◯
Anemia or other blood disease	◯	◯
Cancer	◯	◯
Seizure disorder (epilepsy)	◯	◯
Other neurological disease	◯	◯
Thyroid disease	◯	◯
Osteoarthritis, degenerative arthritis	◯	◯
Back pain	◯	◯
Rheumatoid arthritis	◯	◯

Does it limit your activities?

	No	Yes
Heart disease	◯	◯
High blood pressure	◯	◯
Lung disease	◯	◯
Diabetes	◯	◯
Ulcer or stomach disease	◯	◯
Kidney disease	◯	◯
Liver disease	◯	◯
Anemia or other blood disease	◯	◯
Cancer	◯	◯
Seizure disorder (epilepsy)	◯	◯
Other neurological disease	◯	◯
Thyroid disease	◯	◯
Osteoarthritis, degenerative arthritis	◯	◯
Back pain	◯	◯
Rheumatoid arthritis	◯	◯

Comorbidity Load Score ______________________

Today's date: ______________________

Treatment History

Today's date: ______________________

The following questions ask about any history of receiving treatment for depression, anxiety, or other emotional or mental health problems.

Have you ever seen a therapist, counselor or psychiatrist for emotional or mental health problems?	◯ Yes<br>◯ No
Have you ever been diagnosed with depression?	◯ Yes<br>◯ No
Have you seen a therapist, counselor or psychiatrist for depression, anxiety, or other emotional or mental health problems in the past 4 weeks?	◯ Yes<br>◯ No
Are you currently taking any medications to help you manage depression, anxiety, or other mental health problems?	◯ Yes<br>◯ No

Today's date: ______________________

Routines Survey

Study ID ______________________

Survey 25% Complete

Today's date: ______________________

Below are a few questions asking about routines related to your home and work, the environment where you sleep and how you use your smartphone. Please answer these questions about your routines at this time.

Home Location

What is your current home address?

Street and Number ______________________

What is your current home address?

Apartment or Unit Number ______________________

What is your current home address?

City / Town ______________________

What is your current home address?

State ______________________

What is your current home address?

Zip Code ______________________

Have you temporarily relocated to somewhere other than your home address as a result of COVID-19?

- ◯ Yes
- ◯ No

Employment

What is your current employment status?

- ◯ Employed
- ◯ Unemployed
- ◯ Disability
- ◯ Retired
- ◯ Other
- ◯ Prefer not to answer
- ◯ Student

If Other: ______________________

Has your employment status changed as a result of COVID-19?	◯ Yes<br>◯ No
How many jobs do you have at this time?	◯ 0<br>◯ 1<br>◯ 2<br>◯ 3 or more
At this time, how many hours per week do you work?	__________________________
Which of the following best describes where you work at this time?<br><br>If you are currently not working, please select which option best describes where you spend the majority of your day.	◯ I usually work in one place outside of my home (like an office, store, etc.)<br>◯ I mainly work at home<br>◯ I don't have one place where I work
Has your work location changed as a result of COVID-19?	◯ Yes<br>◯ No
Which best describes your work schedule at this time?<br><br>If you are currently not working, please select which option best describes your daily schedule.	◯ I have a fixed work schedule (e.g. the same hours and days per week)<br>◯ I have a flexible work schedule (the days and hours I work vary)
At this time, In an average workweek, how often do you work from home during your regular work hours?<br><br>If you are currently not working, please select which option best describes the amount of time spent at home during the week.	◯ I don't work from home<br>◯ Less than one day<br>◯ One day<br>◯ 2 days<br>◯ 3 days<br>◯ 4 days<br>◯ 5 days or more
Have your work-from-home hours changed as a result of COVID-19?	◯ Yes<br>◯ No
Have you had to balance child care or other activities in addition to working from home as a result of COVID-19?	◯ Yes<br>◯ No
At this time, on workdays, how often do you travel outside of your primary work location (e.g. errands, visiting other work sites, etc.)?<br><br>If you are currently not working, please select which option best describes how often you leave your primary location.	◯ I don't travel outside of my primary work location<br>◯ Less than one day<br>◯ One day<br>◯ 2 days<br>◯ 3 days<br>◯ 4 days<br>◯ 5 days or more
Do you attend school at present?	◯ Yes<br>◯ No
Are you a full-time or part-time student?	◯ Full-time<br>◯ Part-time

Sleep Habits, Phone & Text Use

Where do you usually keep your phone at night?	◯ Bedroom (or room where you regularly sleep)<br>◯ Room other than where you regularly sleep<br>◯ Prefer not to answer
Do you live alone?	◯ Yes<br>◯ No<br>◯ Prefer not to answer
Has this changed as a result of COVID-19? (i.e., moving to a location with other people living there)	◯ Yes<br>◯ No
Do you share your bedroom with someone?	◯ Yes<br>◯ No<br>◯ Prefer not to answer
Has this changed as a result of COVID-19? (i.e., having to share a bedroom due to relocation)	◯ Yes<br>◯ No
Do you share your home bed with someone else?	◯ Yes<br>◯ No<br>◯ Prefer not to answer
How often do you regularly sleep in a place other than your home?	◯ Almost never (except vacations)<br>◯ 1 to 2 times a week<br>◯ 3 or more times a week<br>◯ Prefer not to answer
If you used text messaging, we'd like to understand your preferences. There are a number of different ways of texting, including using the text messaging application in the phone, as well as text messaging apps that you have downloaded (like WhatsApp, Snapchat, Facebook messenger).<br><br>Which do you use most of the time?	◯ In phone texting<br>◯ Text messaging app (WhatsApp or Snapchat; Facebook messenger; etc.)<br>◯ Prefer not to answer
Do you share your smartphone with anyone?	◯ Yes<br>◯ No
How often does this person (or people) use your phone?	◯ Less than 30 minutes a day<br>◯ 1-5 hours a day<br>◯ 5 or more hours a day
How often do you speak a language other than English in phone calls?	◯ Never<br>◯ Less than once per month<br>◯ 1-2 times per month<br>◯ 3 or more times per month
Today's date:	______________________
QC Confirmation	◯ Yes<br>◯ No

Routines Followup Survey

Record ID ______________________

Survey 63% Complete

Today's date: ______________________

Below are a few questions asking about routines related to your home and work, the environment where you sleep and how you use your smartphone. Please answer these questions about your routines at present.

Home Location

Have you moved since you began this study?
- ◯ Yes
- ◯ No

What is your current home address?

Street and Number ______________________

What is your current home address?

Apartment or Unit Number ______________________

What is your current home address?

City / Town ______________________

What is your current home address?

State ______________________

What is your current home address?

Zip Code ______________________

Have you temporarily relocated to somewhere other than your home address as a result of COVID-19?
- ◯ Yes
- ◯ No

Employment

What is your current employment status?	◯ Employed<br>◯ Unemployed<br>◯ Disability<br>◯ Retired<br>◯ Other<br>◯ Prefer not to answer<br>◯ Student
If Other:	__________________________________

Since you began this study...

Has your employment status changed as a result of COVID-19?	◯ Yes<br>◯ No
How many jobs did you have?	◯ 0<br>◯ 1<br>◯ 2<br>◯ 3 or more
On average, how many hours per week do you work?	__________________________________
Which of the following best describes where you worked?<br><br>If you are currently not working, please select which option best describes where you spend the majority of your day.	◯ I usually work in one place outside of my home (like an office, store, etc.)<br>◯ I mainly work at home<br>◯ I don't have one place where I work
Has your work location changed as a result of COVID-19?	◯ Yes<br>◯ No
Which best describes your work schedule?<br><br>If you are currently not working, please select which option best describes your daily schedule.	◯ I have a fixed work schedule (e.g. the same hours and days per week)<br>◯ I have a flexible work schedule (the days and hours I work vary)
In an average week, how often did you work from home during your regular work hours?<br><br>If you are currently not working, please select which option best describes the amount of time spent at home during the week.	◯ I didn't work from home<br>◯ Less than one day<br>◯ One day<br>◯ 2 days<br>◯ 3 days<br>◯ 4 days<br>◯ 5 days or more
Have your work-from-home hours changed as a result of COVID-19?	◯ Yes<br>◯ No
Have you had to balance child care or other activities in addition to working from home as a result of COVID-19?	◯ Yes<br>◯ No

In an average week, on workdays, how often did you travel outside of your primary work location (e.g. errands, visiting other work sites, etc.)?<br><br>If you are currently not working, please select which option best describes how often you leave your primary location.	◯ I didn't travel outside of my primary work location<br>◯ Less than one day<br>◯ One day<br>◯ 2 days<br>◯ 3 days<br>◯ 4 days<br>◯ 5 days or more
Did you attend school?	◯ Yes<br>◯ No
Were you a full-time or part-time student?	◯ Full-time<br>◯ Part-time

Sleep Habits, Phone & Text Use

Last night, what time did you get into bed?	______________________________
AM or PM?	◯ AM<br>◯ PM
Last night, what time did you try to go to sleep?	______________________________
AM or PM?	◯ AM<br>◯ PM
Last night, how long did it take to fall asleep?	______________________________<br>(minutes)
Last night, what was the total length of your awakenings?	______________________________<br>(minutes)
What time did you wake today?	______________________________
AM or PM?	◯ AM<br>◯ PM
What time did you get out of bed today?	______________________________
AM or PM?	◯ AM<br>◯ PM
Where did you usually keep your phone at night?	◯ Bedroom (or room where you regularly sleep)<br>◯ Room other than where you regularly sleep<br>◯ Prefer not to answer

Today, how many different places did you go outside your home?	◯ 0<br>◯ 1<br>◯ 2<br>◯ 3<br>◯ 4<br>◯ 5<br>◯ 6<br>◯ 7<br>◯ 8<br>◯ >8
Yesterday, how many different places did you go outside your home?	◯ 0<br>◯ 1<br>◯ 2<br>◯ 3<br>◯ 4<br>◯ 5<br>◯ 6<br>◯ 7<br>◯ 8<br>◯ >8
Today, did you exercise?	◯ Yes<br>◯ No
How many minutes did you exercise?	__________________________
Yesterday, did you exercise?	◯ Yes<br>◯ No
How many minutes did you exercise?	__________________________
Did you live alone?	◯ Yes<br>◯ No<br>◯ Prefer not to answer
Has this changed as a result of COVID-19? (i.e., moving to a location with other people living there)	◯ Yes<br>◯ No
Did you share your bedroom with someone?	◯ Yes<br>◯ No<br>◯ Prefer not to answer
Has this changed as a result of COVID-19? (i.e., having to share a bedroom due to relocation)	◯ Yes<br>◯ No<br>◯ Prefer not to answer
Do you share your home bed with someone else?	◯ Yes<br>◯ No
How often did you regularly sleep in a place other than your home?	◯ Almost never (except vacations)<br>◯ 1 to 2 times a week<br>◯ 3 or more times a week<br>◯ Prefer not to answer

If you used text messaging, we'd like to understand your preferences. There are a number of different ways of texting, including using the text messaging application in the phone, as well as text messaging apps that you have downloaded (like WhatsApp, Snapchat, Facebook messenger).<br><br>Which did you use most of the time?	◯ In phone texting<br>◯ Text messaging app (WhatsApp or Snapchat; Facebook messenger; etc.)<br>◯ Prefer not to answer
How often do you speak a language other than English in phone calls?	◯ Never<br>◯ Less than once per month<br>◯ 1-2 times per month<br>◯ 3 or more times per month
Please describe any problems you've been having with the app.	____
Today's date:	________________________________

Holmes-Rahe Stressful Life Events

Study ID ____________________

Mark Y/N whether any of the following items have happened to you during the previous year.

	Yes	No
Death of spouse	○	○
Detention in jail or other institution	○	○
Death of a close family member	○	○
Major personal injury or illness	○	○
Marriage	○	○
Marital reconciliation with mate	○	○
Retirement from work	○	○
Major change in the health or behavior of a family member	○	○
Pregnancy	○	○
Sexual Difficulties	○	○
Gaining a new family member (i.e . ... birth, adoption, older adult moving in, etc.)	○	○
Major business readjustment	○	○
Major change in financial state (i.e . ... a lot worse or better off than usual)	○	○
Death of a close friend	○	○
Changing to a different line of work	○	○
Major change in the number of arguments w/spouse (i.e . ... either a lot more or a lot less than usual 35 regarding child rearing, personal habits. etc.)	○	○
Taking on a mortgage (for home, business, etc . ...)	○	○
Foreclosure on a mortgage or loan	○	○
Major change in responsibilities at work (i.e. promotion, demotion, etc.)	○	○

Son or daughter leaving home (marriage, attending college, joined mil.)	○	○
In-law troubles	○	○
Outstanding personal achievement	○	○
Spouse beginning or ceasing work outside the home	○	○
Beginning or ceasing formal schooling	○	○
Major change in living condition (new home, remodeling, deterioration of neighborhood or home etc.)	○	○
Revision of personal habits (dress manners. associations. quitting smoking)	○	○
Troubles with the boss	○	○
Major changes in working hours or conditions	○	○
Changes in residence	○	○
Changing to a new school	○	○
Major change in usual type and/or amount of recreation	○	○
Major change in church activity (i.e . ... a lot more or less than usual)	○	○
Major change in social activities (clubs, movies, visiting, etc.)	○	○
Taking on a loan (car, tv, freezer, etc.)	○	○
Major change in sleeping habits (a lot more or a lot less than usual)	○	○
Major change in number of family get-tog ethers("")	○	○
Major change in eating habits (a lot more or less food intake, or very different meal hours or 15 surroundings)	○	○
Vacation	○	○
Major holidays	○	○

Minor violations of the law (traffic tickets, jaywalking, disturbing the peace. etc.) ○ ○

Holmes-Rahe Score ______________________

TRAUMA HISTORY QUESTIONNAIRE

The following is a series of questions about serious or traumatic life events. These types of events actually occur with some regularity, although we would like to believe they are rare, and they affect how people feel about, react to, and/or think about things subsequently. Knowing about the occurrence of such events, and reactions to them, will help us to develop programs for prevention, education, and other services. The questionnaire is divided into questions covering crime experiences, general disaster and trauma questions, and questions about physical and sexual experiences.

For each event, please indicate (circle) whether it happened and, if it did, the number of times and your approximate age when it happened (give your best guess if you are not sure). Also note the nature of your relationship to the person involved and the specific nature of the event, if appropriate.

Crime-Related Events		**Circle one**		***If you circled yes, please indicate***	
				**Number of times**	**Approximate age(s)**
1	Has anyone ever tried to take something directly from you by using force or the threat of force, such as a stick-up or mugging?	No	Yes		
2	Has anyone ever attempted to rob you or actually robbed you (i.e., stolen your personal belongings)?	No	Yes		
3	Has anyone ever attempted to or succeeded in breaking into your home when you were not there?	No	Yes		
4	Has anyone ever attempted to or succeed in breaking into your home while you were there?	No	Yes		
General Disaster and Trauma		**Circle one**		***If you circled yes, please indicate***	
				**Number of times**	**Approximate age(s)**
5	Have you ever had a serious accident at work, in a car, or somewhere else? (**If yes**, please specify below)<br>____	No	Yes		
6	Have you ever experienced a natural disaster such as a tornado, hurricane, flood or major earthquake, etc., where you felt you or your loved ones were in danger of death or injury? (**If yes**, please specify below)<br>____	No	Yes		

7	Have you ever experienced a "man-made" disaster such as a train crash, building collapse, bank robbery, fire, etc., where you felt you or your loved ones were in danger of death or injury? (**If yes**, please specify below)<br>_______________________________	No	Yes		
8	Have you ever been exposed to dangerous chemicals or radioactivity that might threaten your health?	No	Yes		
9	Have you ever been in any other situation in which you were seriously injured? (**If yes**, please specify below)<br>_______________________________	No	Yes		
10	Have you ever been in any other situation in which you feared you might be killed or seriously injured? (**If yes**, please specify below)<br>_______________________________	No	Yes		
11	Have you ever seen someone seriously injured or killed? (**If yes**, please specify who below)<br>_______________________________	No	Yes		
12	Have you ever seen dead bodies (other than at a funeral) or had to handle dead bodies for any reason? (**If yes**, please specify below)<br>_______________________________	No	Yes		
13	Have you ever had a close friend or family member murdered, or killed by a drunk driver? (**If yes**, please specify relationship [e.g., mother, grandson, etc.] below)<br>_______________________________	No	Yes		
14	Have you ever had a spouse, romantic partner, or child die? (**If yes**, please specify relationship below)<br>_______________________________	No	Yes		
15	Have you ever had a serious or life-threatening illness? (**If yes**, please specify below)<br>_______________________________	No	Yes		
16	Have you ever received news of a serious injury, life-threatening illness, or unexpected death of someone close to you? (**If yes**, please indicate below)<br>_______________________________	No	Yes		

17	Have you ever had to engage in combat while in military service in an official or unofficial war zone? (**<u>If yes</u>**, please indicate where below)<br>____	No	Yes		
Physical and Sexual Experiences		**Circle one**		***If you circled yes, please indicate***	
				**Repeated?**	**Approximate age(s) and frequency**
18	Has anyone ever made you have intercourse or oral or anal sex against your will? (**<u>If yes</u>**, please indicate nature of relationship with person [e.g., stranger, friend, relative, parent, sibling] below)<br>____	No	Yes		
19	Has anyone ever touched private parts of your body, or made you touch theirs, under force or threat? (**<u>If yes</u>**, please indicate nature of relationship with person [e.g., stranger, friend, relative, parent, sibling] below)<br>____	No	Yes		
20	Other than incidents mentioned in Questions 18 and 19, have there been any other situations in which another person tried to force you to have an unwanted sexual contact?	No	Yes		
21	Has anyone, including family members or friends, ever attacked you with a gun, knife, or some other weapon?	No	Yes		
22	Has anyone, including family members or friends, ever attacked you <u>without</u> a weapon and seriously injured you?	No	Yes		
23	Has anyone in your family ever beaten, spanked, or pushed you hard enough to cause injury?	No	Yes		
24	Have you experienced any other extraordinarily stressful situation or event that is not covered above? (**<u>If yes</u>**, please specify below)<br>____	No	Yes		

Citation:

Hooper, L. M., Stockton, P., Krupnick, J., & Green, B. L. (2011). The development, use, and psychometric properties of the Trauma History Questionnaire. *Journal of Loss and Trauma, 16,* 258-283.
