# Supplementary material for: Investigating low intensity focused ultrasound pulsation in anhedonic depression—A randomized controlled trial
Source: Front Hum Neurosci. 2025 Mar 24;19:1478534. doi: 10.3389/fnhum.2025.1478534 (PMC11973349; doi:10.3389/fnhum.2025.1478534)
Supplement: Supplementary file 2 [file Data_Sheet_2.pdf]

Supplementary Methods

S1. Ecological Momentary Assessment (EMA)

The EMA is a 3-part, 19-item assessment that measures current mood and experiences. This EMA battery was developed by Zachary Cohen, Amelia Welborn, & Michelle Craske in consultation with Eiko Fried, Gabi Khazanov, and Courtney Forbes (Cohen, Forbes, Khazanov, & Fried, in prep; see https://osf.io/6xnv2/). We'd also like to thank Aaron Fisher, Samir Akre, and Kerneau Seok for helpful conversations when developing this EMA. Some items were drawn from the SCOUT study (Kirtley et al., 2024), the WARN-D study (Fried et al., 2023), and the ESM Item Repository (Kirtley et al., 2018).

The first block comprises 8 mood items (presentation order internally randomized) asked in the form of "How XYZ are you right now?" and rated using a 5-point Likert scale: [0] Not at all; [1] A little bit; [2] Moderately; [3] Quite a bit; [4] Extremely. We tried to balance the number of positive and negative items, resulting in the following 8 items: Sad, Stressed, Anxious, Annoyed/Irritated, Energetic, Happy, Motivated, Engaged.

The second block comprises 6 true/false questions (presentation order internally randomized) intended to assess reward-related constructs (thoughts/emotions/behaviors): "Right now…"

1. I'm looking forward to an upcoming activity
2. I'm feeling good after doing something
3. I'm putting effort into planning something that will be enjoyable in the future
4. I could be doing something positive but am not because I don't think I'd enjoy it
5. I could be doing something positive but am not because it feels like too much effort
6. I'm feeling a sense of meaning and purpose

These items were developed to align with contemporary frameworks for anhedonia and reward-processing domains (e.g., anticipatory vs. consummatory), with the 6th question added to assess eudaimonic well-being.

The third block comprises two questions each for social contacts and activities. First, participants are asked to indicate who they are with: "Right now, I am with (includes phone/video/digital) choose all that apply:"

1. Friend
2. Family
3. Romantic partner
4. Co-workers/classmate/acquaintance
5. Stranger
6. Pet
7. Other
8. No one

Then, unless the participant selected “no one” (in which case this question is skipped), the participant rates how much they are enjoying this company on a 7-point (1:7) Likert scale (“I am enjoying this company” with text only on the anchors: 1= “Not at all”; 7 = “Very much”).

Next, the participants were asked to indicate their current activity: “Right now (before you opened this survey), I am doing (choose all that apply):”
1. Social activity (offline or online)
2. Physical activity (e.g. gym, sports)
3. Active leisure activity (hobby, board game)
4. Passive leisure activity (e.g. watching TV, scrolling Instagram)
5. Working/studying
6. Tasks (e.g. cleaning house)
7. On my way to somewhere
8. Eating
9. Childcare
10. Other
11. Nothing

And finally, participants were asked to rate their enjoyment of the current activity (“I am enjoying what I’m doing”) on the same 7-point Likert scale described above (from “not at all” to “very much”).

The rationale for the specific ordering of these three blocks was as follows: We wanted participants to answer the mood questions first so that their responses would not be affected by the process of reflecting on their (e.g.,) thoughts/activities during blocks 2 and 3. Similarly, we wanted participants’ “general” responses to questions from block 2, and our concern was that if we asked those after block 3, that their responses to block 2 might be overly focused on the social interactions or activities we ask about in block 3.

For OPTIMA participants, during the OPTIMA study EMA are completed 5x a day in three 8-day bursts sent at baseline, week 6, and at the end of the study (“pre-scan”, roughly week 12; which serves as the “baseline” EMA assessment for the ILIAD study). For non-OPTIMA participants in ILIAD, their baseline EMA data is collected 5x a day for the 15 days prior to the first LIFUP session. All ILIAD participants subsequently complete 1x daily EMA from the day of LIFUP 1 through the day of LIFUP 3, and 8 days of 5x daily EMAs starting 7 days prior to scan 2 through the day of scan. If scan 2 is <8 days after LIFUP 3, the 5x daily EMAs will override the 1x daily EMAs. An additional 8 days of 5x daily EMA data is collected during Week 5 of the ILIAD protocol. Finally, a single instance of the EMA question set was included alongside the PHQ-14/PVSS for all timepoints at which these retrospective self-report assessments were administered. For the 5x per day EMA, ping timing is linked to 5 evenly spaced anchors (2 hours and 12 minutes apart): 10:12 am (T1); 12:24 pm (T2); 2:36 pm (T3); 4:48 pm (T4); and 7:00 pm (T5). Specific ping timing was randomized +/- up to 15 minutes around those 5 anchors using a random draw from a uniform distribution, and participants were informed they had up to 30 minutes after they received their EMA to finish responding (with reminders automatically

delivered for incomplete EMA at 15 minutes and 5 minutes). This timing was selected to try and avoid the first (early morning) ping arriving during breakfast/commute, to avoid the final ping coming in too late at night, and to avoid any overlap between EMA responses.

S2. Eudaimonic Well-Being Questionnaire (EWBQ)

The Eudaimonic Well-Being Questionnaire (EWBQ) is a brief 5-item self-report measure that was originally adapted by Chrissy Sandman (Sandman, 2023) and then further adapted by Sandman, Cohen, and Craske (for the purpose of the OPTIMA/ILIAD studies) as a past-week assessment of trait-like eudaimonic well-being in the areas of meaning, purpose, values, relationships, and self-image. It draws from two existing questionnaires: the 14-item Mental Health Continuum Short Form (MCH-SF)(Keyes, 2006; Keyes et al., 2008) and the Daily Meaning Scale (DMS)(Steger et al., 2008). The EWBQ uses the same 5-point Likert scale used in this study’s EMA mood items ([0] Not at all; [1] A little bit; [2] Moderately; [3] Quite a bit; [4] Extremely), and asks participants: “Using the 0-4 scale below, indicate the extent to which you have felt the following ways **over the past week.**”

1. How much do you feel your life has a sense of meaning?
2. How much do you feel a sense of purpose in life?
3. How much do you feel connected to your values?
4. How much do you feel like you have warm and trusting relationships with others?
5. How much do you feel confident and positive about yourself?

The EWBQ’s first two items were adapted from the DMS’ (Steger et al., 2008) two items (‘’how meaningful does your life feel?’’ and ‘’how much do you feel your life has purpose?’’), and based on the MCH-SF item for purpose in life, worded “During the past month, how often did you feel (0=Never to 5=Everyday) that your life has a sense of direction or meaning to it”, with the goal of harmonizing item wording/structure consistent across all 5 questions and making the items more trait-like than state-like. EWBQ Item 3 was created by Sandman due to lack of established measures that assess present moment awareness of connection of current behavior – or memory – to values (Barney et al., 2019). Item 4 of the EWBQ was adapted directly from the MCH-SF psychological well-being item for Positive Relationships (connection to others), worded: “During the past month, how often did you feel that you had warm and trusting relationships with others.” Finally, EWBQ Item 5 was adapted from the MCH-SF Self-Acceptance item (“How much do you feel like you like most parts of your personality?”).

S3. Modified SITBI-R+CSSRS-SR

The Modified SITBI-R + CSSRS-SR (MSC-SR) [adapted by Zachary Cohen, Amelia Welborn, and Michelle Craske, in consultation with Kathryn Fox and Shirley Wang] is a lifetime and current suicidality measure that combines and adapts items from the CSSRS-SR (Posner et al., 2011) and the SITBI-R (Fox et al., 2020). A number of items from CSSRS-SR and SITBI-R were

excluded from the combined measure in an attempt to decrease respondent burden. The combined measure is a 22-item self-report assessment of current and past experiences of nonsuicidal self-injury, suicidal gestures, suicidal ideation, intent, plan, and attempts. The measure yields two severity scores: lifetime severity (maximum score 11) and current severity (maximum score 10). Higher scores indicate greater severity. See Yarrington et al., (in prep) for more details[1].

S4. Modified Patient Health Questionnaire – 14-item version

Patient Health Questionnaire - 14 item version (PHQ-14): The PHQ-14 is a modified version of the Patient Health Questionnaire, originally developed by Kroenke, Spitzer and Williams (Kroenke et al., 2001) as a 9-item self-report screener (PHQ-9) assessing depression symptoms over the past 2-weeks. The PHQ-9 is one of the most widely used depression screening questionnaires, especially in psychiatric and primary care settings (Costantini et al., 2021). As part of the Depression Symptom Response Project, Zachary Cohen, Daniel Cohen, and Eiko Fried developed modified versions of the PHQ[2] that are better-suited for longitudinal studies tracking week-to-week changes in specific symptoms.

The version used in this study is a “past week” version of the PHQ-14, developed for conducting more frequent assessments (instead of the classic administration that asks about the past 2 weeks). In the “classic” past two weeks version, the response options (anchors) are: [0] Not At All; [1] Several Days; [2] More Than Half the Days; [3] Nearly Every Day. However, when asking about the past week, the option “Several Days” (i.e., 3-4 days) would be overlapping with “More Than Half the Days” (technically >3.5), so for the “past week” version we replaced the “Several Days” anchor with “Less than Half the Days” (“less” was chosen instead of “fewer” after asking more than a dozen individuals and determining that “less than” sounded more natural next to “more than”).

Next, to cover a broader range of symptoms consistent with other scales such as the IDS-30 (Rush et al., 1996), and in part due to our strong interest in understanding the value of digital phenotyping (DP), we created a dis-aggregated version that separates the four items from the classic PHQ-9 that describe compound symptoms: Item 2 (“Feeling down, depressed or hopeless”) was separated into “Feeling down or depressed” (sad mood) and “Feeling hopeless” (hopelessness); Item 3 (“Trouble falling asleep, staying asleep, or sleeping too much”) was separated into “Trouble falling asleep or staying asleep” (roughly early-insomnia and middle-insomnia) and “Sleeping too much” (hypersomnia); Item 5 (“Poor appetite or overeating”) was separated into “Poor appetite” or “Overeating”; and Item 8 (“Moving or speaking so slowly that other people could have noticed. Or, the opposite – being so fidgety or restless that you have been moving around a lot more than usual”) was separated into “Moving or speaking so slowly that other people could have noticed” (psychomotor retardation) and “Being so fidgety or restless that you have been moving around a lot more than usual” (psychomotor agitation). As is

[1] https://osf.io/zm2ta/?view_only=2401267adb92493180c1fe08c0cb120c
[2] Manuscript in preparation; see https://osf.io/w4rj9/ for more details and copies of the modified versions

often done in research studies, the suicidality item (Q9) was removed, and two additional depression symptoms of interest that are assessed in other commonly used measures (Fried, 2017) were added: irritability (“Feeling irritable”) and libido (“Little interest in sex”).

Because we expected this modified version would often be administered alongside the GAD-7 (Spitzer et al., 2006), we also tweaked the instructions by removing the words “any of” from the original instructions ("Over the last 2 weeks, how often have you been bothered by any of the following problems?") so that the prompt was better-aligned with the instructions of the GAD-7. Finally, the phrase “reading the newspaper” in item 7 of the PHQ-9, (“Trouble concentrating on things, such as reading the newspaper or watching television”), was changed to just “reading” to increase the applicability of this item. In order to recreate a total score that approximates the PHQ-8 total score (the PHQ-8 is simply the PHQ-9 administered without the suicidality question [Q9]), the original items are summed alongside the max value from the dis-aggregated compound symptom pairs (see table S1 below).

In multiple samples (some collected specifically for psychometric validation of this modified PHQ) in which participants have filled out the original PHQ-8 and then been asked to separately rate the dis-aggregated symptoms, the original PHQ-8 total score and the “pseudo” PHQ-8 total score derived from taking the max value response from the compound symptoms have been correlated between r = .96 to r = .98 (manuscript in preparation; see https://osf.io/w4rj9/ for more details). The new items for irritability and libido are not included in calculation of the overall depression severity score for the PHQ-14; instead, these items are intended to be utilized for separate symptom-specific analyses.

Supplementary Tables

Table S1. The relationship between PHQ-14 and PHQ-8 questions and scoring.

Original PHQ-8 Items	PHQ14 Q #	PHQ-14 Item #	Equation	Relationship between PHQ-8 and PHQ-14
1. Little interest or pleasure in doing things	Q1	1	1	Identical
2. Feeling down, depressed or hopeless	Q2 | Q3	2a | 2b	max(2a,2b)	Separated sad mood and hopelessness
3. Trouble falling asleep, staying asleep, or sleeping too much	Q4 | Q5	3a | 3b	max(3a,3b)	Separated “early-insomnia + middle-insomnia” and “hypersomnia”
4. Feeling tired or having little energy	Q6	4	4	Identical
5. Poor appetite or overeating	Q7 | Q8	5a | 5b	max(5a,5b)	Separated reduced vs increased appetite/eating
6. Feeling bad about yourself – or that you’re a failure or have let yourself or your family down	Q9	6	6	Identical
7. Trouble concentrating on things, such as reading the newspaper or watching television	Q10	7	7	Identical* (*almost - I removed "the newspaper" to make more contemporary)
8. Moving or speaking so slowly that other people could have noticed. Or, the opposite – being so fidgety or restless that you have been moving around a lot more than usual	Q11 | Q12	8a | 8b	max(8a,8b)	Separated psychomotor retardation and agitation

Table S2. Timing of Assessments. *Only administered to non-OPTIMA participants. **Only administered to OPTIMA participants. EMA and MRI schedules are omitted from this table as they have variable timing that does not fit within the time points described in this table. See figure 1, sections 3.2.1 and 3.3 in the main manuscript, and supplemental methods S1 for more information regarding the timing of these elements of the protocol. The Edinburgh Handedness Inventory and MRI screening form are both eligibility assessment measures and therefore were not included in Table 2 of main manuscript. The partial re-administration of the TestMyBrain neurocognitive battery during week 6 includes only the Multiracial Emotion Identification and Gradual Onset Continuous Performance Test.

	Time	W0*	Pre-Scan*	Completed During OPTIMA Protocol**	Baseline Assessments **	W1							W2	W3	W4	W5
**Study Protocol Timing**		D-14 – D-8	D-7 – D-1		D-4 – D-0	D1	D2	D3	D4	D5	D6	D7	D8-14	D15-21	D22-28	D29-35
**LIFUP**						x		x		x						
**PHQ-9**	3 min						x		x		x		x		x	
**PHQ-14 (past week)**	3 min	x	x		x	x							x	x	x	x
**PVSS-21 (past week)**	2 min	x	x		x	x							x	x	x	x
**Edinburgh Handedness Inventory**	1 min		x	x												
**MRI Screening Form**	5 min		x	x									x			
**Prescribed Medications**	15 min		x	x												
**Demographics Questionnaire**	5 min		x	x												
**QIDS**	2 min		x		x											x
**BITe Survey**	1min		x		x											x
**MSC-SR**	2 min		x	x												x
**GAD-7**	1 min		x		x											x
**WSAS**	2 min		x		x											x
**WHO-5 Wellbeing Index**	1 min		x		x											x
**PSQI**	5 min		x		x											x
**SHAPS**	1 min		x		x											x
**AMI**	2 min		x		x											x
**RRS-brooding subscale - state**	2 min		x		x											x
**RRS-brooding subscale - trait**	2 min	x														

**EWBQ**	1 min		x		x											x
**USDA Housing/ Food Insecurity**	1 min		x	x												
**Comorbidities Questionnaire**	1-2 min		x	x												
**Treatment History Questionnaire**	1 min		x	x												x
**Routines**	4 min		x													
**Holmes Rahe Life Stress Survey**	10 min		x		x											x
**Trauma History Questionnaire**	5 min		x	x												
**SAGE-SR**	20 min		x	x												
**TestMyBrain neurocognitive battery**	40 min		x	x												x (partial)
**A/V Recorded interview**	15 min		x (scan day)		x											

Table S3. Digital phenotyping data collected with Apple SensorKit.

SensorKit Data	
**Category**	**Variables Collected**
**Accelerometer**	acceleration
**Rotation Rate**	rotationRate
**Ambient Light Sensor**	chromaticity, lux, placement
**On Wrist State**	crownOrientation, onWrist, wristLocation
**Pedometer**	numberOfSteps, distance, averageActivePace, currentPace, currentCadence, floorsAscended, floorsDescended
**Visits**	arrivalDateInterval, departureDateInterval, distanceFromHome, locationCategory
**Keyboard Metrics**	duration, keyboardIdentifier, version, width, height, inputModes, totalWords, totalAlteredWords, totalTaps, totalDrags, totalDeletes, totalEmojis, totalPaths, totalPathTime, totalPathLength, totalAutoCorrections, totalSpaceCorrections, totalRetroCorrections, totalTranspositionCorrections, totalInsertKeyCorrections, totalSkipTouchCorrections, totalNearKeyCorrections, totalSubstitutionCorrections, totalHitTestCorrections, totalTypingDuration, totalPathPauses, totalPauses, totalTypingEpisodes, SRKeyboardMetrics.ProbabilityMetric, touchDownUp, spaceTouchDownUp, deleteTouchDownUp, shortWordCharKeyTouchDownUp, touchDownDown, charKeyToPrediction, shortWordCharKeyToCharKey, charKeyToAnyTapKey, anyTapToCharKey, spaceToCharKey, charKeyToSpaceKey, spaceToDeleteKey, deleteToSpaceKey, spaceToSpaceKey, spaceToShiftKey, spaceToPlaneChangeKey, spaceToPredictionKey, deleteToCharKey, charKeyToDelete, deleteToDelete, deleteToDeletes, deleteToShiftKey, deleteToPlaneChangeKey, anyTapToPlaneChangeKey, planeChangeToAnyTap, charKeyToPlaneChangeKey, planeChangeKeyToCharKey, deleteToPath, pathToDelete, spaceToPath, pathToSpace, pathToPath, longWordTouchDownUp, longWordTouchDownDown, pathTypingSpeed, typingSpeed, longWordUpErrorDistance, longWordDownErrorDistance, upErrorDistance, downErrorDistance, spaceUpErrorDistance, spaceDownErrorDistance, deleteUpErrorDistance, deleteDownErrorDistance, shortWordCharKeyUpErrorDistance, shortWordCharKeyDownErrorDistance, pathErrorDistanceRatio, wordCount, emojiCount, SentimentCategory
**Device Usage Report**	duration, totalScreenWakes, totalUnlocks, totalUnlockDuration, applicationUsageByCategory, bundleIdentifier, usageTime, categoryKey, notificationUsageByCategory, notificationUsage, webUsageByCategory, webUsage
**Message Usage Report**	duration, totalIncomingMessages, totalOutgoingMessages, totalUniqueContacts
**Phone Usage Report**	duration, totalIncomingCalls, totalOutgoingCalls, totalPhoneCallDuration, totalUniqueContacts

Table S4. Digital phenotyping data collected with Apple HealthKit

HealthKit Data	
**Category**	**Variables Collected**
**Characteristic Identifiers**	activityMoveMode, biologicalSex, bloodType, dateOfBirth, fitzpatrickSkinType
**Activity**	stepCount, distanceWalkingRunning, distanceCycling, pushCount, distanceWheelchair, swimmingStrokeCount, distanceSwimming, distanceDownhillSnowSports, basalEnergyBurned, activeEnergyBurned, flightsClimbed, nikeFuel, appleExerciseTime, appleStandHour, appleStandTime, vo2Max, lowCardioFitnessEvent
**Body Measurement**	height, bodyMass, bodyMassIndex, leanBodyMass, bodyFatPercentage, waistCircumference
**Reproductive Health**	basalBodyTemperature, cervicalMucusQuality, contraceptive, intermenstrualBleeding, lactation, menstrualFlow, ovulationTestResult, pregnancy, sexualActivity, pregnancyTestResult, progesteroneTestResult
**Hearing**	environmentalAudioExposure, headphoneAudioExposure, environmentalAudioExposureEvent, headphoneAudioExposureEvent
**Vital Signs**	heartRate, lowHeartRateEvent, highHeartRateEvent, irregularHeartRhythmEvent, restingHeartRate, heartRateVariabilitySDNN, walkingHeartRateAverage, HKDataTypeIdentifierHeartBeatSeries, HKElectrocardiogramType, oxygenSaturation, bodyTemperature, bloodPressure, bloodPressureSystolic, bloodPressureDiastolic, respiratoryRate
**Nutrition**	food, HKMetadataKeyFoodType, dietaryEnergyConsumed, dietaryCarbohydrates, dietaryFiber, dietarySugar, dietaryFatTotal, dietaryFatMonounsaturated, dietaryFatPolyunsaturated, dietaryFatSaturated, dietaryCholesterol, dietaryProtein, dietaryVitaminA, dietaryThiamin, dietaryRiboflavin, DietaryNiacin, dietaryPantothenicAcid, dietaryVitaminB6, dietaryBiotin, dietaryVitaminB12, dietaryVitaminC, dietaryVitaminD, dietaryVitaminE, dietaryVitaminK, dietaryFolate, dietaryCalcium, dietaryChloride, dietaryIron, dietaryMagnesium, dietaryPhosphorus, dietaryPotassium, dietarySodium, dietaryZinc, dietaryWater, dietaryCaffeine, dietaryChromium, dietaryCopper, dietaryIodine, dietaryManganese, dietaryMolybdenum, dietarySelenium
**Alcohol Consumption**	bloodAlcoholContent, numberOfAlcoholicBeverages
**Electrocardiogram**	classification, averageHeartRate, symptomStatus, numberOfVoltageMeasurements, samplingFrequency, VoltageMeasurement, Lead

References

Barney, J. L., Lillis, J., Haynos, A. F., Forman, E., and Juarascio, A. S. (2019). Assessing the valuing process in Acceptance and Commitment Therapy: Experts' review of the current status and recommendations for future measure development. *J Contextual Behav Sci* 12, 225–233.

Costantini, L., Pasquarella, C., Odone, A., Colucci, M. E., Costanza, A., Serafini, G., et al. (2021). Screening for depression in primary care with Patient Health Questionnaire-9 (PHQ-9): A systematic review. *J. Affect. Disord.* 279, 473–483.

Fried, E. I. (2017). The 52 symptoms of major depression: Lack of content overlap among seven common depression scales. *J. Affect. Disord.* 208, 191–197.

Fried, E. I., Proppert, R. K. K., and Rieble, C. L. (2023). Building an Early Warning System for Depression: Rationale, Objectives, and Methods of the WARN-D Study. *Clin Psychol Eur* 5, e10075.

Keyes, C. L. M. (2006). The Subjective Well-Being of America's Youth: Toward a Comprehensive Assessment. *Adolesc. Fam. Health* 4, 3–11.

Keyes, C. L. M., Wissing, M., Potgieter, J. P., Temane, M., Kruger, A., and van Rooy, S. (2008). Evaluation of the mental health continuum-short form (MHC-SF) in setswana-speaking South Africans. *Clin. Psychol. Psychother.* 15, 181–192.

Kirtley, O. J., Claes, S., Schoefs, S., Portzky, G., and Myin-Germeys, I. (2024). Investigating social interactive pathways between exposure to suicide attempts and suicidal thoughts and behaviours in young adults: The Social Connections amOng yoUth in disTress (SCOUT-Clinical) Project. Available at: https://osf.io/aqh4k/

Kirtley, O. J., Hiekkaranta, A. P., Kunkels, Y., Eisele, G., Verhoeven, D., Nierop, M. V., et al. (2018). The experience sampling method (ESM) item repository. doi: 10.17605/OSF.IO/KG376

Kroenke, K., Spitzer, R. L., and Williams, J. B. (2001). The PHQ-9: validity of a brief depression severity measure. *J. Gen. Intern. Med.* 16, 606–613.

Rush, A. J., Gullion, C. M., Basco, M. R., Jarrett, R. B., and Trivedi, M. H. (1996). The Inventory of Depressive Symptomatology (IDS): psychometric properties. *Psychol. Med.* 26, 477–486.

Sandman, C. (2023). Upregulating Positive Affect Through Imaginal Recounting in Anhedonia. University of California, Los Angeles.

Spitzer, R. L., Kroenke, K., Williams, J. B. W., and Löwe, B. (2006). A brief measure for assessing generalized anxiety disorder: the GAD-7. *Arch. Intern. Med.* 166, 1092–1097.

Steger, M. F., Kashdan, T. B., and Oishi, S. (2008). Being good by doing good: Daily eudaimonic activity and well-being. *J. Res. Pers.* 42, 22–42.
